# Genome Sequencing and Comparative Analysis of *Saccharomyces cerevisiae* Strains of the Peterhof Genetic Collection

**DOI:** 10.1371/journal.pone.0154722

**Published:** 2016-05-06

**Authors:** Polina B. Drozdova, Oleg V. Tarasov, Andrew G. Matveenko, Elina A. Radchenko, Julia V. Sopova, Dmitrii E. Polev, Sergey G. Inge-Vechtomov, Pavel V. Dobrynin

**Affiliations:** 1 Dept. of Genetics and Biotechnology, Saint Petersburg State University, St. Petersburg, Russia; 2 Bioinformatics Institute, St. Petersburg, Russia; 3 St. Petersburg Scientific Center of RAS, St. Petersburg, Russia; 4 St. Petersburg Branch, Vavilov Institute of General Genetics of the Russian Academy of Sciences, St. Petersburg, Russia; 5 Laboratory of Amyloid Biology, Saint Petersburg State University, St. Petersburg, Russia; 6 Institute of Translational Biomedicine, Saint Petersburg State University, St. Petersburg, Russia; 7 Research Resource Center for Molecular and Cell Technologies, Research Park, Saint-Petersburg State University, St. Petersburg, Russia; 8 Theodosius Dobzhansky Center for Genome Bioinformatics, Saint Petersburg State University, St. Petersburg, Russia; University of Leicester, UNITED KINGDOM

## Abstract

The Peterhof genetic collection of *Saccharomyces cerevisiae* strains (PGC) is a large laboratory stock that has accumulated several thousands of strains for over than half a century. It originated independently of other common laboratory stocks from a distillery lineage (race XII). Several PGC strains have been extensively used in certain fields of yeast research but their genomes have not been thoroughly explored yet. Here we employed whole genome sequencing to characterize five selected PGC strains including one of the closest to the progenitor, 15V-P4, and several strains that have been used to study translation termination and prions in yeast (25-25-2V-P3982, 1B-D1606, 74-D694, and 6P-33G-D373). The genetic distance between the PGC progenitor and S288C is comparable to that between two geographically isolated populations. The PGC seems to be closer to two bakery strains than to S288C-related laboratory stocks or European wine strains. In genomes of the PGC strains, we found several loci which are absent from the S288C genome; 15V-P4 harbors a rare combination of the gene cluster characteristic for wine strains and the *RTM1* cluster. We closely examined known and previously uncharacterized gene variants of particular strains and were able to establish the molecular basis for known phenotypes including phenylalanine auxotrophy, clumping behavior and galactose utilization. Finally, we made sequencing data and results of the analysis available for the yeast community. Our data widen the knowledge about genetic variation between *Saccharomyces cerevisiae* strains and can form the basis for planning future work in PGC-related strains and with PGC-derived alleles.

## Introduction

*Saccharomyces cerevisiae* is a widely used model organism. The S288C strain is the ancestor to many commonly used yeast laboratory strains [1, 2] and provided the first eukaryotic genome to be completely sequenced [3]. S288C and related strains originate from the Carbondale breeding stock of C. Lindegren [4], which resulted from crosses between different strains of *S. cerevisiae* as well as other *Saccharomyces* species [1, 5]. To date, genomes of more than 150 yeast strains of different origins have been sequenced [6–8]. Comparison of such a variety of genomes helps to clarify the natural history of yeast populations and allows to identify genomic elements that are selected under specific conditions. Strains distant from S288C may provide new insights in various fields of yeast genetics as it was demonstrated in studies of genetic control of metabolism and chromosome recombination [9–13]. The Peterhof genetic collection (PGC) contains several strains that became widely used in the field of translation termination ([14–16], and other works) and yeast prion research ([17–19], and other works).

The PGC originates from a Russian industrial distillery lineage (“race XII”) that is thought to be distant from the populations that gave rise to the S288C lineage. In contrast to S288C and most known laboratory strains, the progenitor strain of the PGC is presumed to derive from a single yeast population [20]. The collection is maintained with separate registration of diploids obtained from mating of strains ascending to the progenitor (designated with capital ‘P’ and a consecutive number) and those of hybrid (mosaic) origin (designated with ‘D’ or some other letter in a similar way), and thus enables tracing of ancestors of any strain [21].

A number of genetic variations between Peterhof and S288C-derived strains has been identified, but whole genome data for this big collection of strains are scarce. Thus, we aimed to characterize the genomes of several PGC strains.

## Results and Discussion

### Origin of the strains

In this work, we analyzed genomes of five PGC-related *S. cerevisiae* strains. The PGC came from the initial industrial lineage XII with low ascospore viability (*ca*. 0.7%) through 7 generations of intratetrad self-fertilization. The resulting heterothallic diploid strain with high ascospore viability (*ca*. 90%), XII_7_, should be considered the *bona fide* progenitor of the Peterhof lineage of strains. A haploid prototroph selected after 3 more inbred crosses, 15V-P4, gave rise to the core part of the collection; thus, strains ascending only to 15V-P4 and XII_7_ are considered pure Peterhof strains [20, 22]. Apart from 15V-P4, we analyzed genomes of four haploid laboratory strains. 25-25-2V-P3982 (the full name of the strain is 25-25-dU8-132-L28-2V-P3982) [23] is a laboratory strain of a presumably pure Peterhof origin, while 1B-D1606 [15], 74-D694 [24], and 6P-33G-D373 Asp^+^[25, 26] descend from hybrids between Peterhof and S288C-derived strains (further referred to as strains of hybrid origin). The pedigree of strains selected for whole genome sequencing is shown in S1 Fig. For brevity, these strains are henceforth referred to as 25-25, 1B, 74, and 6P-33G; however, as these are not legitimate strain names, we strongly discourage from using these shortened names while mentioning these strains elsewhere.

Genomes of these strains were of particular interest for a number of reasons. 25-25, 1B, and 6P-33G are derived from strains that have been widely used to study termination of translation, and these or closely related strains were the source of all sequenced PGC alleles of the translation termination factor genes as well as auxotrophy markers [15, 16, 27–29]. These sequences are instrumental in validating the quality of the next generation sequencing results. 74 and its derivatives have been exploited to study the [*PSI*^+^] prion [24], and genomic data for a [*PSI*^+^] variant of this strain was published earlier [30]. 6P-33G was particularly interesting because it has been recently shown to be disomic for chromosome VIII [26] and could therefore serve as a control in copy number variation analysis. 1B and 6P-33G are closely related (S1 Fig), which might provide material to study recombination patterns. Finally, these strains had a number of phenotypes lacking known molecular basis (see below).

### Genome assembly and gene annotation

Raw reads produced with either Ion Torrent PGM (15V-P4, 25-25, 1B, and 6P-33G) or with Illumina GAII (74) were assembled *de novo* (Table 1). The resulting assemblies were characterized by varying quality as assessed by Quast [31], with the best results for the 1B genome and the worst for the 6P-33G genome. Quality of assemblies produced with Ion Torrent data increased according to the coverage, while Illumina data for the 74 genome produced a lower quality assembly despite higher coverage, which was probably due to shorter reads. Contigs were scaffolded to produce pseudochromosomes using the S288C genome as a reference.

**Table 1:** *De novo* assembly statistics.

Strain	15V-P4	25-25	1B	74	6P-33G
Number of contigs*	1,165	891	480	1,514	3,039
Largest contig, bases*	86,918	122,204	252,839	71,636	22,054
Total length, bases*	11,666,974	11,614,012	11,567,449	11,330,585	10,013,534
N50*	19,288	26,385	72,884	11,948	4,341
S288C genome fraction, %*	91.87	92.78	94.17	92.53	76.01
Median S288C genome coverage	19x	22x	35x	42x	16x
Number of S288C genes found (%) **	5,115 (87%)	5,000 (85%)	4,993 (84%)	5,058 (86%)	4,004 (68%)
Complete CEGMA core genes found (%)	241 (97%)	240 (97%)	243 (98%)	243 (98%)	210 (85%)
Partial CEGMA core genes found (%)	244 (98%)	241 (97%)	243 (98%)	243 (98%)	226 (91%)

* Calculated with Quast.

** Calculated as one-to-one orthologs with ProteinOrtho clustering.

The assemblies were annotated with several *de novo* and alignment-based gene finders, and then united annotations were obtained with the MAKER2 pipeline [32]. Genes found with this pipeline were mapped to the known S288C genes with ProteinOrtho [33] clustering. For all the genomes excluding 6P-33G we were able to find at least about 85% of reference genome genes. We also assessed quality of assemblies with CEGMA [34] and were able to find almost 100% of common eukaryotic core genes in all assemblies except for 6P-33G and over 90% in all genomes. These results signify that the assemblies can be used in downstream analyses.

### Origin of the PGC in a phylogenetic context

Since the PGC was established independently from the Carbondale breeding stock [4, 20], we were interested in determining the phylogenetic relationships of the PGC progenitor and other *S. cerevisiae* strains. To assess this, we applied two complementary approaches.

**Fig 1:**
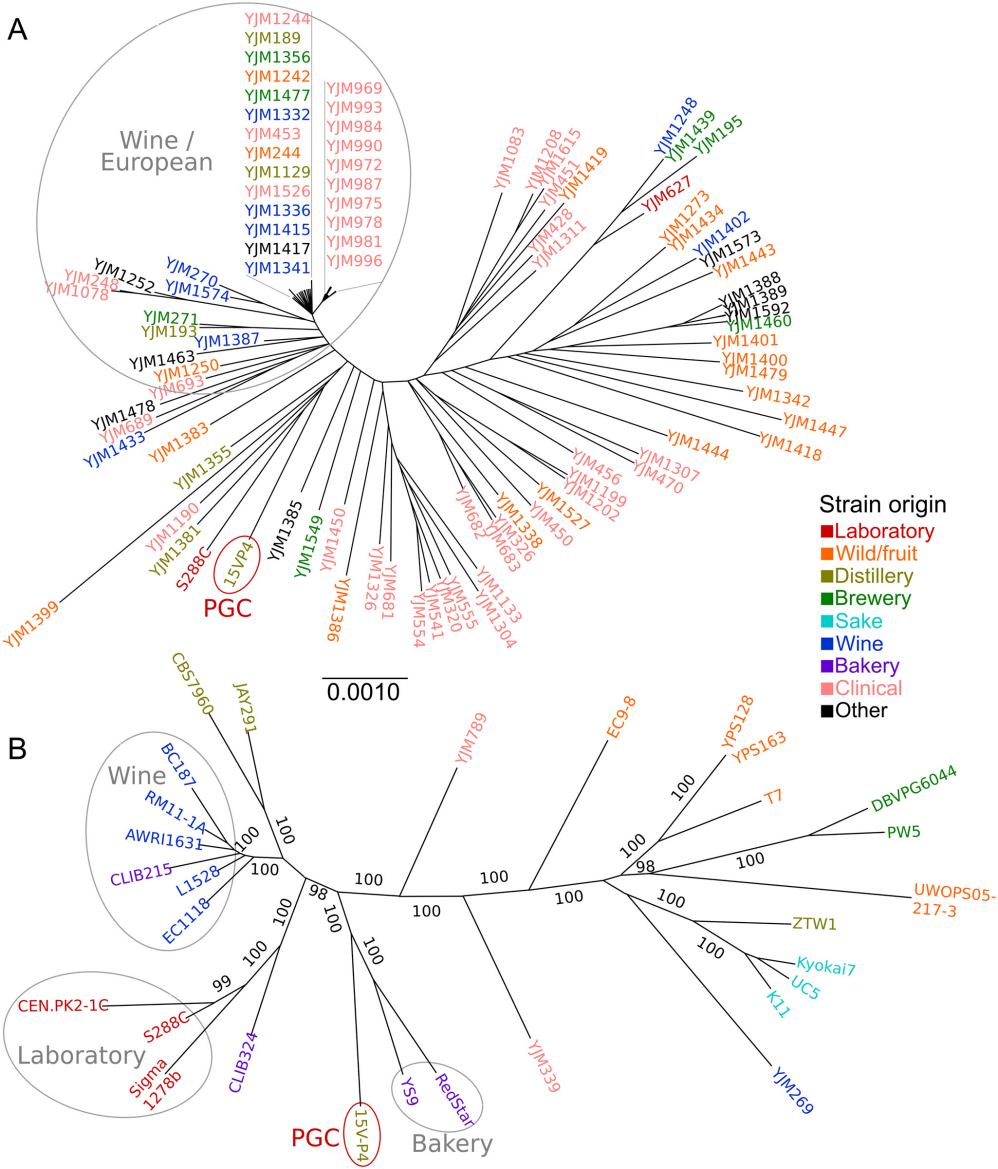
15V-P4 position in the phylogeny of *S. cerevisiae* strains. (A) Neighbor joining phylogenetic tree of 95 strains including 15V-P4 inferred from alignment of conservative chromosome regions. (B) Phylogenetic tree of 29 strains including 15V-P4 inferred from sequences of 807 common genes under the GTR+G model and tested with 500 bootstrap replicates. Branch bootstrap values greater than 95 are indicated. In both trees, strain names are colored according to functional origin. Grey circles highlight either the population group (A) or common functional origin (B). Branch lengths are given in the same scale on both trees. PGC, the Peterhof genetic collection.

First, we used the largest *S. cerevisiae* tree presently available, which is based on a set of highly conserved regions from all nuclear chromosomes [8]. We extracted the corresponding sequences from the 15V-P4 genome assembly and constructed an alignment with 217,304 positions from 95 genomes to re-infer the phylogenetic tree (Fig 1A). The overall tree topology is similar to that reported originally [8]. According to this tree, the closest strains to 15V-P4 are YJM1190, YJM1381, YJM1399, S288C and YJM1355. Even though these strains are of different geographic origins, two of them, YJM1355 and YJM1381, are of distillery origin [8], similar to 15V-P4. In addition, we merged SNV data for 15V-P4 with the available dataset [8] and assessed the population ancestry of the PGC with STRUCTURE [35, 36]. 15V-P4 appeared to possess an admixed genome with most probable ancestry to wine and human-associated populations (S2A Fig), as well as strains close to it on the tree.

As these data did not elucidate the PGC origin, we turned to an alternative approach. We sampled coding sequences of 15V-P4 and 28 strains of different origin from the *Saccharomyces* Genome Database (SGD). Only genomes with more than 3500 genes annotated were selected for the analysis, and 807 genes were found in all of them. Total alignment based on sequences of these genes (S1 Table) included 852,372 nucleotide positions. The resulting phylogenetic tree inferred from common ORFs (Fig 1B) is generally similar in topology to those inferred previously from total genomic SNVs or non-reference ORFs [7, 37]. The tree shows a major clade including three groups: the first one uniting common laboratory strains (*e.g.* S288C), the second comprising commercial wine and bioethanol strains, and the third consisting of 15V-P4 and two bakery strains, YS9 and RedStar (Fig 1B). We added the latter two strains to the SNV-based 95-genomes tree and confirmed this result, as 15V-P4 was closer to YS9 and RedStar than to S288C (S2B Fig). Thus, the distillery lineage ancestral to the PGC might have itself originated from a bakery strain.

### Non-reference genes in PGC genomes

Newly sequenced *S. cerevisiae* strains are frequently found to contain genes absent from the genome of the reference strain (see [7]). To determine whether the Peterhof strains possess such genes, we divided all the annotated genes found into known (*i.e.* those found in the reference genome) and novel (non-reference) ones. The list of novel genes was used as a BLAST query, and the BLAST output was manually curated; presence of genes from other strains or species was re-confirmed with Exonerate protein2genome search for the best BLAST hit against genome assemblies. We found a total of 11 non-reference genes in the 15V-P4 genome (Table 2); some of these genes were inherited by the other strains.

**Table 2:** Genes absent from the S288C genome but found in 15V-P4.

Gene name	Protein function	S288C	15V-P4
*AMI1-A*	Amidase	No	Yes
*KHR1*	Killer toxin	No	Yes
*RTM1*	Lipid exporter	No	Yes
*SCY_1426*	Hypothetical zinc finger transcription factor	No	Yes
SUCX*	Invertase	*SUC2* only	≥ 2 *SUCX* genes
Wine12	5-oxo-L-prolinase	No	Pseudogene?
Wine23	Nicotinic acid permease	No	Yes
Wine34	Flocculin	No	Yes
Wine45	Transcription factor	No	Yes
Wine56	Transcription factor	No	Yes

Yes/No denotes presence/absence of the corresponding gene.

* Members of the *SUC* family, see S2 Table.

All five strains studied possess the *KHR1* gene, which encodes a killer toxin of unknown nature [38]. In 25-25 and 1B, this gene was annotated on the same contigs as known chromosome IX genes, which corroborates findings of Wei *et al.* [39], who localized this gene on chromosome IX in the YJM789 strain.

All the strains analyzed except for 74 possess the *RTM1* gene annotated on its own contig. It was identified in a BLAST search as *S. bayanus*, *S. carlsbergensis*, or *S. pastorianus* *rtm1* and then re-confirmed with Exonerate search for the Rtm1 protein sequence from YJM789 (Genbank accession EDN59063.1). *RTM1* encodes a lipid-translocating exporter and is known to be advantageous for strains growing on molasses [40, 41]. The *RTM1* gene is a member of a subtelomeric three-gene locus found in several clinical, industrial, and environmental isolates [40]. In the strains harboring *RTM1*, the same contig contains the second member of this cluster encoding a *ca.* 750 amino acid long hypothetical zinc finger transcription factor.

The *RTM1* cluster is usually found in association with genes of the *SUC* (sucrose utilization) family [40]. The *SUC* genes of *S. cerevisiae* fall into two categories, either *SUC2* (YIL162W) or others found in subtelomeric regions [42]. The S288C strain possesses the *SUC2* gene but not the subtelomeric *SUC* genes. Analysis of the reads aligned to the region of chromosome IX corresponding to the *SUC2* (YIL162W) ORF revealed at least two different *SUC* genes (S2 Table), although we were unable to determine their exact number. This finding agrees with recently reported presence of *SUC2*, *SUC5*, and *SUC8* in the XII_7_ strain (parental to 15V-P4) revealed with DNA-DNA hybridization of PFGE-resolved chromosomes with a *SUC2* probe [43].

In 15V-P4, but not in the rest of the strains, we also found the so-called ‘wine cluster’ consisting of five genes (Wine12–Wine56, see Table 2) initially identified in wine strains [44]. Sequence analysis suggests that the 5-oxo-L-prolinase gene (Wine12) is a pseudogene as it contains two frameshifts while the other four genes may be active. Interestingly, 15V-P4 appears to be the first non-wine yeast strain reported to obtain simultaneously the *RTM1* cluster and the wine-specific cluster; genomes sequenced so far rarely contain both clusters [7, 45]). Wine cluster is supposed to move within yeast genomes easily, therefore it could be lost quickly during laboratory breeding [44].

In addition, we found a *Saccharomyces* *pastorianus* amidase gene *AMI1-A*(Uniprot A9CMR9) on its own contig in 15V-P4 but not in any other PGC strain. We also detected this gene in several other *S. cerevisiae* genomes (*e.g.*, RedStar and Kyokai7).

Thus, we showed that the PGC progenitor possesses a unique combination of non-reference genes; however, other PGC strains lost many of them which is presumably a common effect of a laboratory breeding.

In addition to non-reference genes, we looked for regions that could have been introgressed into the 15V-P4 genomes from closely related *Saccharomyces* species. We employed two alternative methods, search for ORFs that are more similar to one of the available *Saccharomyces sensu stricto* genomes than to S288C and alignment of 15V-P4 short reads to concatenated *S. sensu stricto* genomes. In the first analysis, we did not find any regions covering the whole gene and being more similar to a non-*cerevisiae* genome (S3 Table). In the second analysis, the overall alignment of 15V-P4 was very similar to S288C and dissimilar to YJM248 [8], a positive control for introgression (S3 Fig). Thus, we could not reliably identify any introgressed regions, and this result argues against possible interspecific hybridization in the original distillery lineage.

### Copy number variations in PGC genomes

**Fig 2:**
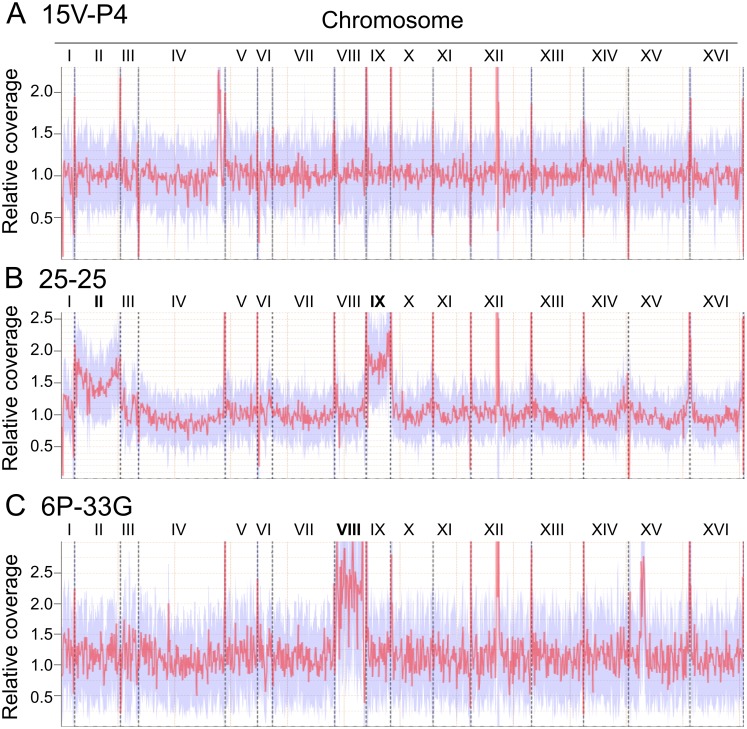
Genome coverage across reference for representative strains. (A) 15V-P4, (B) 25-25, (C) 6P-33G. Dashed lines signify chromosome borders.

Genome content variations such as chromosomal rearrangements and aneuploidy were found in different *S. cerevisiae* strains [8, 46, 47]. We exploited reference genome coverage to estimate relative sequencing depth of each chromosome. It was mostly uniform for 15V-P4, 1B and 74 (Fig 2A, S4 Fig) but not for 25-25 and 6P-33G. Interestingly, a region on the right arm of chromosome IV seemed to be duplicated in 15V-P4, as well as a region of the left arm of chromosome XV in 6P-33G. In the 25-25 genome, chromosomes II and IX had higher coverage than the others (Fig 2B), which suggests that the population of this strain includes a significant number of aneuploid cells. In case of 6P-33G, chromosome VIII coverage was about 2-fold higher compared to the other chromosomes (Fig 2C). This finding perfectly agrees with the earlier reported data on chromosome VIII disomy in this strain [26].

Next, we used the mrCaNaVaR pipeline to analyze possible segmental duplications or deletions more precisely (Table 3). For full list of the regions annotated as amplified or deleted, see S4 Table. In accordance to the reference genome coverage data, much more regions were annotated as amplified in 25-25 and 6P-33G genomes. This tendency becomes even clearer if the numbers of genes included into the amplified regions are compared (about 150 in euploid strains and about 400 to 500 in strains with a tendency to aneuploidy; see S5 Table). 101 amplified genes were common in the amplified regions of all five genomes but almost all of them have close paralogs and may thus represent false positive findings. We conclude that the results of analysis of amplified regions are very noisy and should be interpreted with caution. There are at least two possible reasons, the great number of recently amplified genes in yeast due to the whole genome duplication in the lineage leading to *S. cerevisiae* [48, 49] and presence of aneuploid strains in our analysis.

**Table 3:** Lengths of regions annotated as amplified or deleted in each strain and counts of genes included into each of these regions.

Strain	15V-P4	25-25	1B	74	6P-33G
Total length of amplified regions, bp	458,407	856,981	474,369	613,763	1,054,510
Total number of amplified genes	179	392	141	159	499
Total length of deleted regions, bp	84,475	53,117	39,722	24,334	36,913
Total number of deleted genes	20	9	5	6	24

Analysis of deleted regions should not be prone to such noise. Importantly, we were able to confirm all known whole-ORF deletions, *i.e. URA3* deletion in 25-25, *HIS3* deletion in 74 and *SUP35* deletion in 6P-33G (see S5 Table, and S1 Appendix). In addition, we looked for other deleted genes. Two genes, *FLO10* and *NFT1*, were presumably deleted in all the strains. These genes are adjacent on the right arm of chromosome XI, and their absence might indeed represent a common feature of PGC-related strains.

### Single nucleotide variations

In order to assess the difference between Peterhof strains and the reference strain S288C, we aligned short reads to the S288C genome. Typically, about 95% of reads were aligned. Then, we called single nucleotide variations (SNVs), and filtered out low quality differences and differences in repeat regions.

**Fig 3:**
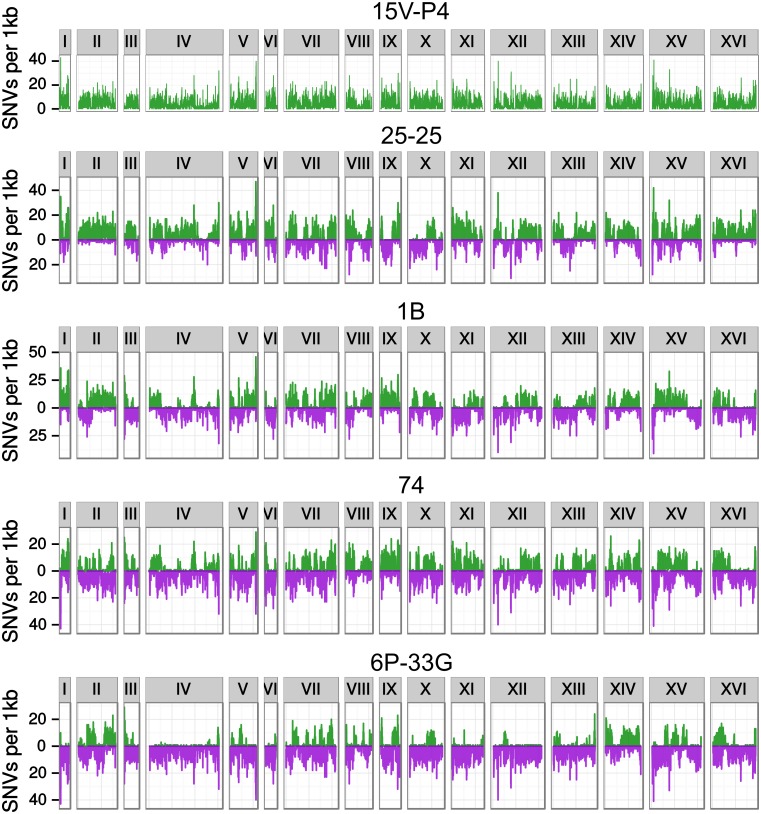
Distribution of variable sites shown in chromosomal coordinates of S288C. Green: SNVs compared to S288C. Purple: SNVs compared to 15V-P4. Each chromosome is framed.

First, we analyzed the distribution of substitutions in the ancestor strain of the PGC. The distribution of polymorphic sites in 1 kb windows across the S288C chromosomes seemed quite uniform (Fig 3, upper panel). Functional classification of substitutions performed with SNPeff enabled us to find 97 nonsense, 10675 missense, and 18534 silent mutations, as well as 16020 intergenic variations. It directly translates to dN/dS = 0.58 hinting at presence of selection pressure.

Then, we estimated the number of short indels compared to reference in each of the genomes analyzed. The Ion Torrent technology is prone to errors in homopolymer regions [50, 51]. However, these errors are random and should not reproduce in all the reads aligned to a particular position. Thus, we filtered only indels supported by all reads aligning to this position (100% supported indels) as they are less likely to represent sequencing errors. Unlike the total number of indels, the number of 100% supported indels was roughly proportional to the number of SNVs (S6 Table), which consolidates our approach.

Many strains of the Peterhof genetic collection are known to be of hybrid origin, *i.e.* to originate from at least two yeast genetic lines, Peterhof and Carbondale breeding stocks. Using 15V-P4 as the reference Peterhof strain and S288C as a common reference strain, we called all variations between each strain and two reference strains. The results of this analysis are presented at Fig 3. As expected, strains ascending to ‘D’ diploids showed long tracts of either non-Peterhof or non-S288C substitutions, as we would expect for a mosaic genome. Surprisingly, the same kind of analysis for the 25-25 strain indicates that it has hybrid origin even though it was previously described as a pure Peterhof strain [23].

We estimated genetic difference between PGC strains and S288C as the number of pairwise SNVs using the genome of S288C as a common reference and neighbor joining algorithm (S5 Fig). 15V-P4 and S288C differ by 45,842 SNVs which is comparable to the level of divergence of about 50,000 SNVs between distant *S. cerevisiae* populations reported previously [6]. As expected, the 25-25 strain is the most similar to 15V-P4. However, these two strains have much more pairwise SNVs than we expected, which supports the idea that this strain should have had a non-Peterhof ancestor. 1B and 74 are roughly half as distant from S288C as from 15V-P4; this result is consistent with their known hybrid origin. 6P-33G appears to be closer to S288C than to 15V-P4.

### Selected SNVs and associated phenotypes

Since a number of genetic and phenotypic differences between particular Peterhof strains and S288C-derived strains had been identified previously ([15, 27] and other works), we employed these data in our analysis by looking for already known variations. This search served two purposes. First, we used it to validate our pipeline. Second, as variations in strains close to the PGC progenitor (*e.g.*, 15V-P4) have never been analyzed, this approach enabled us to assess whether known differences converge to the common ancestor of the Peterhof genetic collection or were attained during the laboratory breeding of the strains.

We searched the Peterhof strains for the known genetic variations in several selectable marker genes. The whole genome sequencing results conform to the previous data and complete the missing information about precise mapping of some mutations (Table 4; S1 Appendix).

**Table 4:** Selectable marker mutations in the PGC strains.

Allele	Known change	Variations detected in this study*	Strains
*ade1-14*	G732A (→TGA) [52–54], G554A [54]	G732A (→TGA)	25-25, 74, 1B
*ade2-144,717* (*ade2-144,791*)**	Nonsense (*ade2-144*) [55] + downstream non-suppressible (*ade2-717* = *791*) [56] mutation	T422A + G423A (→TAA), C1517T (→P506L)	6P-33G
*his3Δ200*	Deletion (-205 to +172) [57]	Deletion (-205 to +172)	74
*his7-1*	A229T (→TAA) [58, 59]	A229T (→TAA)	25-25, 6P-33G, 1B
*leu2-B2* (*leu2-1*, *leu2-1A*, *leu2-01*)**	Unidentified non-suppressible mutation	G748A (→D250N)	25-25
*leu2-3,112*	249insG, 792insG [60]	249insG, 792insG	74, 1B
*lys2-87* (L28)**	Nonsense mutation, TGA [52]	G3465A (→TGA)	25-25
*lys9-A21*	T605A (→TAA) [58, 59]	T605A (→TAA)	6P-33G, 1B
*pheA10* (*pha2P-A10*)***	Nonsense mutation [22], TAA [61]	A481T (→TAA)	6P-33G
*thr4-B15*	Nonsense mutation, TGA [52]	A1180T (→TGA)	25-25
*trp1-289*	C403T (→TAG) [59, 62]	C403T (→TAG)	6P-33G, 74, 1B
*ura3Δ* (dU8)	Complete deletion of *URA3* (unpublished data)	Deletion (-188 to +76)	25-25
*ura3-52*	Ty1 insertion (transcribing left to right) at 121 [63]	Ty insertion at 121****	6P-33G, 74, 1B

Nucleotide positions in 5’ UTR are preceded with the minus sign while those in 3’ UTR with the plus sign; numbers indicate distance from the beginning or the end of the ORF, respectively. Stop codon type or amino acid substitution are indicated after an arrow for mutations that must lead to known auxotrophic phenotypes.

* Only differences from the corresponding wild type alleles are listed. For complete list of substitutions, see S1 Appendix.

** Synonymous designations.

*** Assigned to the *PHA2* locus in this work.

**** Includes duplication of insertion flanking sequence (GTACC).

Some PGC strains have been extensively used to obtain large collections of strains with suppressor mutations in release factor genes *SUP35* (*SUP2*) and *SUP45*(*SUP1*) [15, 16, 28, 64]. Their sequences were previously identified in dU8-132-L28-2V-P3982 and 1B, respectively [15, 27]. We detected all the mutations we were aware of (S1 Appendix). In 15V-P4, we found all the SNVs identified previously in wild type Peterhof *SUP35* and *SUP45* alleles. Thus, we proved that these alleles had been inherited from the common ancestor of the PGC.

#### PHA2

6P-33G, as well as its direct ancestor, 33G-D373, is known to bear a phenylalanine auxotrophy mutation *pheA10* [65]. This mutation has been shown to be a TAA nonsense, as it was suppressible by ochre suppressors ([61] and unpublished data) but has never been mapped to a particular gene (S1 Appendix). So, we looked for mutations in phenylalanine biosynthesis genes and found a premature termination codon (PTC) in the *PHA2* gene (see S6 Fig and S1 Appendix for details).

**Fig 4:**
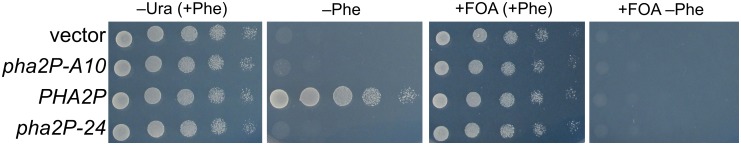
Only *PHA2P* but not *pha2* mutant alleles compensate for the *pheA10* phenylalanine auxotrophy. 33G-D373 was transformed with plasmids bearing indicated *PHA2* alleles. Series of 5-fold dilutions on synthetic media are shown. Vector, pRS316.

To find whether this nonsense mutation in *PHA2* is responsible for the phenylalanine auxotrophy we cloned either the wild type PGC allele *PHA2P* or the mutant allele (designated as *pha2P-A10*) into a centromeric *URA3* vector. Introduction of *PHA2P*, but not *pha2P-A10*-containing plasmid into 33G-D373 restored growth on media lacking phenylalanine (S6C Fig). Furthermore, loss of the plasmid-borne *PHA2P* allele on 5-FOA-containing medium led to immediate loss of phenylalanine prototrophy (Fig 4). We also obtained a *pha2* double missense mutation (*pha2P-24*) which was unable to compensate for phenylalanine auxotrophy in 33G-D373 (S6C Fig, Fig 4). Thus, not only *pha2P-A10*, but other defects in *PHA2P* may lead to a phenylalanine auxotrophy, which is consistent with previous findings [66] and supports *pha2* usefulness as a selectable marker. We also showed that level of *pha2P-A10* suppression is higher in Asp^-^ than in Asp^+^ derivative of 6P-33G (S6D Fig), consistent with comparative levels of suppression of other nonsense mutations in the two derivatives [26]. Thus, this allele might also be employed to study nonsense suppression in yeast.

At the next step, we looked for novel nonsense mutations as their effect is the easiest to predict. We found a total of 16 to 78 genes with PTCs in the Peterhof strains (5 of these genes were common for all 5 strains) and 2 genes, *FLO8* and *CRS5*, in which stop codons present in S288C were absent from PGC strains (S7 Table). Among those, we further investigated absence of a PTC in *FLO8* and presence of a PTC in *MSN4*.

#### Clumping

**Fig 5:**
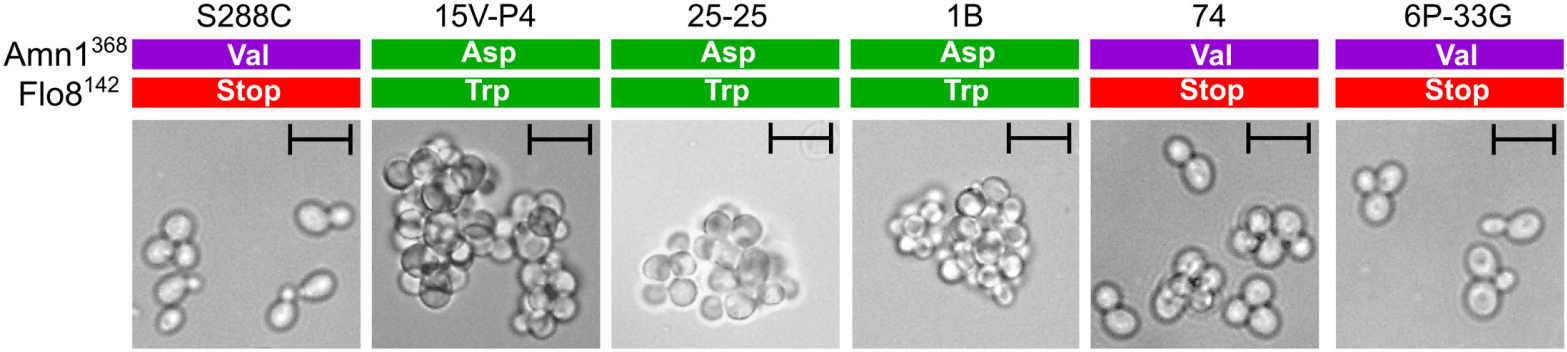
Cell aggregation phenotypes of strains analyzed correlate with *AMN1* and *FLO8* alleles. The scale bar indicates 10 um. Amn1 and Flo8 variants are shown in color (green: associated with “clumping” phenotypes; red and purple: “non-clumping”). Representative microphotographs out of five fields of view of yeast liquid medium cultures in early stationary phase are shown.

Cells of Peterhof-derived strains tend to clump together in liquid medium (unpublished data). Cell aggregation is a very complex trait but some genes contributing to its control are known. Flo8 is a transcription factor contributing much in flocculation, diploid filamentous growth, and haploid invasive growth in yeast [67–70]. Several PGC-related diploid strains were shown to form pseudohyphae on solid medium and to contain the *FLO8* allele encoding the full length protein [71, 72]. Amn1 is another transcriptional regulator with a clear link between the sequence variant and the cell aggregation phenotype [70]. S288C and closely related strains with Amn1^368Val^ and Flo8^142Stop^ do not form clumps, while variants Amn1^368Asp^ and Flo8^142Trp^ contribute much to the change from non-clumping to clumping phenotype [68, 70]. We observed the same tendency in PGC strains: those with Amn1^Asp368^ and Flo8^Trp142^ showed clear clumping phenotype while those with known loss-of-function variants were much less prone to form cell aggregates (Fig 5). Unfortunately, we could not assess the effect of the two variable positions separately.

We also addressed the suppressibility of the *flo8* stop codon by two modifiers of translation termination, the [*PSI*^+^] prion and the Asp^+^ determinant. We found no difference in clumping efficiency between isogenic strains with different suppressor phenotypes (data not shown). This is consistent with previous data showing almost complete absence of the *flo8* stop codon bypass [73].

#### MSN4

The *MSN4* gene encodes a transcription factor with many targets including heat shock proteins. A PTC in *MSN4* in the 74 genome was first attested in Fitzpatrick *et al.* [30]. As we used the same data, our analysis produced the same result. In addition, we found the same mutation in 25-25 and 15V-P4. Thus, the other two strains probably inherited this substitution from 15V-P4.

To test whether this mutation has any associated phenotype, we cloned *MSN4* into a centromeric vector pRS316 and transformed 74 with this construct. As slight difference in thermotolerance was shown for [*PSI*^+^] and [*psi*^-^] derivatives of 74 [74], we exploited both strains. We could not see any change in thermotolerance upon plasmid addition (S7 Fig).

#### Multiple substitutions in the *GAL* locus

Several PGC strains, including 1B, are Gal-, *i.e.* they manifest no growth on media containing galactose (even with raffinose) as a sole carbon source (unpublished data). We found that in 1B and 6P-33G lengthy regions of chromosome II, which include the *GAL* locus, are enriched in different sets of SNVs which are neither 15V-P4- nor S288C-derived. We suppose that these regions may have been inherited from some ancestors other than S288C or 15V-P4; therefore, the comparison of the *GAL* locus sequences may provide additional information on genealogy of PGC strains.

The *GAL* locus encodes three enzymes of galactose metabolism (Gal7, Gal10, and Gal1). To determine possible origin of this locus in PGC strains and mutation(s) causing the galactose utilization defect in 1B, we compared the *GAL* locus sequences (chromosome II from 274,427 to 280,607) of 5 PGC genomes and 38 strains from SGD. The *GAL* locus of 6P-33G seemed to be identical to that of JK9-3d, SEY6210, and YPH499, the ancestor of the other two strains (S8A Fig) [75, 76]. YPH499 originates from a strain congenic to S288C [77] but is known to have some non-S288C SNVs [78]. In 1B, the *GAL* locus was almost identical to that of D273-10B (S8A Fig) and FL100 strains which have common origin from F. Sherman’s lab [79, 80]. Together, these data imply possible lineages of laboratory yeast strains that might have left their footprints in the history of PGC.

The only SNV unique for 1B is a missense mutation *GAL10*^C287T^ (Gal10^Ala96Val^) (S8A Fig). D273-10B and FL100 are known to be Gal+ [81, 82]; therefore, this substitution may be responsible for the Gal- phenotype of 1B. To test this assumption, we transformed 1B with plasmids containing the complete *GAL* locus of S288C or its fragments and found that only the plasmids containing *GAL10* reverted 1B to Gal+ (S8B Fig). Gal10 has an UDP-galactose-4-epimerase (GALE) activity [83]. The residue 96^Ala^ in *S. cerevisiae* Gal10 (93^Ala^ in human GALE) is located in highly conservative NAD and UDP-hexose binding pocket [84, 85]. In human GALE, substitution of the adjacent 94^Val^ with Met (S8C Fig) leads to severe galactosemia [86, 87]. Thus, we presume that Gal10^A96V^ is associated with inability to utilize galactose as a carbon source.

### Conclusions

The Peterhof genetic collection (PGC) of yeasts is an almost unique example of a laboratory stock developed independently of the Carbondale breeding stock (S288C-related strains) and including several thousands various strains which can be used in different types of experiments. We have characterized genomes of five PGC strains and made the data available for the yeast community. It allowed us to investigate the phylogenetic relationship of PGC strains with other *S. cerevisiae* strains. Interestingly, phylogenetic analysis places the progenitor strain, 15V-P4, together with two bakery strains even though it originates from a distillery lineage.

SNV analysis showed that the genetic difference between the progenitor strain of PGC and S288C is approximately the same as the difference between distant yeast populations reported earlier [6]. Importantly, the genetic distances between the strains generally are in good agreement with their pedigree. However, new data imply that one presumably pure Peterhof strain, 25-25, is of hybrid origin.

Strains of PGC possess several loci absent from S288C. None of these loci are unique for PGC strains but their combination such as in 15V-P4 has not been reported yet. To the extent of our knowledge, it is the first sequenced non-wine strain with *RTM1* and wine clusters at the same time.

We were able to find out the exact sequence differences corresponding to most previously known phenotypes. Particularly, we mapped the *pheA10* nonsense mutation to the *PHA2* gene and identified a missense mutation in *GAL10* as the reason behind galactose utilization defect in 1B. We also found and validated some genetic variations providing insight into physiological differences between PGC and S288C-derived strains. We saw very good agreement between allelic states of *FLO8* and *AMN1* with cell clumping pattern. Dissimilar to commonly used S288C-based laboratory strains, Peterhof strains can be used to study aggregation phenotypes and pseudohyphal growth [71, 72], and our data further support this usage.

Together, our data widen the knowledge about genetic variation between *Saccharomyces cerevisiae* strains, link some previously known phenotypes to newly identified sequence differences and form the basis for planning future work in PGC-related strains and with PGC-derived alleles.

## Materials and Methods

### Yeast strains

Yeast strains used in this work are listed in the Table 5 and are available upon request at the Department of Genetics and Biotechnology of the Saint Petersburg State University.

**Table 5:** Yeast strains used in this work.

Name	Known genotype	References
15V-P4	*MAT* **a** *prototroph*	[20]
25-25-2V-P3982* (25-25)	*MAT* **a** *ade1-14 his7-1 lys2-87 ura3Δ leu2-B2 thr4-B15 sup35-25 sup45-400*	[23]
6P-33G-D373 Asp^+^ (6P-33G)	*MAT*α pha2P-A10 (pheA10) ade2-144,717 his7-1 lys9-A21 trp1-289 ura3-52 leu2-3,112sup35Δ::PmSUP35-LEU2** *CUP1//CUP1 (chromosome VIII disomy)*	[25, 26]
6P-33G-D373 Asp^-^	*MAT*α pha2P-A10 (pheA10) ade2-144,717 his7-1 lys9-A21 trp1-289 ura3-52 leu2-3,112 sup35Δ::PmSUP35-LEU2**	[25, 26]
33G-D373	*MAT*α *pha2P-A10 (pheA10) ade2-144,717 his7-1 lys9-A21 trp1-289 ura3-52 leu2-3,112*	[65]
74-D694	*MAT* **a** *ade1-14 trp1-289 his3Δ200 ura3-52 leu2-3,112* [*psi*^-^] [*PIN*^+^]	[24]
P-74-D694***	*MAT* **a** *ade1-14 trp1-289 his3Δ200 ura3-52 leu2-3,112* [*PSI*^+^] [*PIN*^+^]	K. Volkov, unpublished; [61, 89]
1B-D1606	*MAT*α ade1-14 his7-1 lys9-A21 trp1-289 ura3-52 leu2-3,112 gal10-1B****	[15]
S1 (isogenic to S288C)	*MAT*α *gal2 flo8-1 hap1*	[1, 8]

* The full name of the strain is 25-25-dU8-132-L28-2V-P3982.

** *PmSUP35*, *Pichia methanolica SUP35*.

*** A [*PSI*^+^] derivative of 74-D694. In addition, other [*PSI*^+^] derivatives of 74-D694 (OT55 and OT56) [61, 88] and their [*psi*^-^] isolates were used for the clumping experiments.

**** *gal10-1B* designates the *gal10^C287T^* allele from 1B-D1606 discovered in this work.

### DNA extraction and genome sequencing

Raw reads for the genome of 74-D694 [*PSI*^+^] variant originating from Yury Chernoff lab were produced with Illumina GAII [30] and downloaded from http://bioinf.nuim.ie/wp-content/uploads/2011/10/74D_sequence.txt.zip. Single end libraries for genomes of 15V-P4, 25-25-2V-P3982, 1B-D1606, and 6P-33G-D373 Asp^+^ strains were sequenced with Ion Torrent PGM^™^ machine. Raw reads are available at the NCBI Sequence Read Archive [PRJNA296913, SRP064279].

DNA extraction was performed with mechanical disruption of yeast cells as described in [89]. YPD was supplemented with 100 to 250 mg/L adenine in case of *ade1* and *ade2* mutant strains.

Genomic DNA library was prepared using Ion Plus Fragment Library Kit, according to the manufacturers recommendations (User Guide Publication Number 4471989, Revision N). Template-positive particles for genomic DNA sequencing were prepared using Ion PGM^™^ Template OT2 400 Kit according to the user guide (Publication number MAN0007218, revision 3.0). Sequencing was conducted using Ion PGM^™^ Sequencing 400 Kit and Ion 318^™^ Chip v2, following the manufacturer’s user guide (Publication Number MAN0007242, Revision 2.0). Sanger sequencing was performed with ABI Prism 3500xl.

All sequencing reactions were performed at the Research Resource Center for Molecular and Cell Technologies of the Saint Petersburg State University.

### Data analysis

Quality control of reads was performed with FastQC [90]. Trimming of reads was performed with fastx_toolkit v0.0.13.1 [91] and cutadapt [92]. Trimming length was chosen according to the basic statistics calculated with FastQC [90].

*De novo* genome assembly was performed with SPAdes [93] v3.1.0 with IonHammer (option—iontorrent for homopolymer correction) for Ion Torrent data and with SPAdes v3.6.0 for Illumina data. Reference-assisted assembly of scaffolds was performed with chromosomer [94]. The S288C genome (Release R64-1-1, downloaded from the *Saccharomyces* Genome Database [95]) was used as a reference throughout this work. Quality of assemblies was estimated with Quast [31] and CEGMA [34].

Genome assemblies were annotated with Exonerate 2.2.0 with protein2genome and est2genome models using sets of S288C proteins and mRNAs, and the following *de novo* gene finders: Augustus 3.0.3 [96], GeneMark-ES v4.21 [97], and SNAP (version 2013-11-29) [98].

All sets of annotations were united using the MAKER2 pipeline v2.31.8 [32]. Genes found with this pipeline were divided into novel and known ones with an in-house Python3 script. This script employs a renaming table produced with proteinortho [33] by clustering the whole sets of ORFs from the reference strain (S288C) and the strain analyzed. RepeatMasker v4.0.5 [99] and Tandem Repeat Finder v4.07 [100] were used to identify and mask repeated sequences.

Introgression analysis was carried out in two ways. First, we looked for S288C ORFs for which the best BLAST hit in the 15V-P4 genome had < 96% identity, extracted the corresponding regions from the 15V-P4 genome and looked for better (> 98% identity) BLAST hits in *S. sensu stricto* genomes, as described by Strope *et al.*[8]. Second, short reads for the 15V-P4, YJM248 and S288C genomes were aligned to a reference consisting of concatenated *S. kudriavzevii* ZP 591, *S. bayanus var. uvarum* CBS 7001, *S. cerevisiae* S288C, *S. kudriavzevii* IFO1802^T^, *S. mikatae* IFO1815^T^, *S. paradoxus* CBS432, *S. eubayanus* FM1318, and *S. arboricolus* H-6 genomes, similarly to the analysis reported in [7]. Depth of coverage was reported with qualimap v2.1 [101]. YJM248 ([8]; GenBank accessions CP004414, CP004618, CP006335, CP004664, CP004758, CP004894, CP005198, CP006123, CP004986, CP005118, CP005295, CP006439, CP005396, CP005498, CP005592, CP006215, CP006478, CP004505, and SRA accession SRR800768) was used as a positive control for introgression in both cases. *S. sensu stricto* assemblies, reported in [102], were retrieved from saccharomycessensustricto.org. Short reads for S288C were reported in [103] and retrieved from SRA (SRR027936). *S. arboricolus* and *S. eubayanus* genome sequences, reported in [104, 105], respectively, were retrieved from the NCBI Genome portal (40941, 577061).

Mapping of short reads to the reference genome was performed with Bowtie v2.1.0 [106] for analysis of single nucleotide variation and with mrFast [107] for analysis of copy number variation. Quality control of bam files was performed with qualimap v2.2 [101]. Alignments were visualized with UGENE [108, 109] for manual check.

SNV calling on alignments was performed with samtools [110] v1.0 mpileup command with subsequent filtering of low quality (q < 30) and low coverage (DP < 3) positions with vcftools [111] v1.0. Heterozygous indels and variations in the repeat regions were also filtered out.

SNVs were annotated with snpEff 4.1 [112]. snpEff output was used to infer the effect of mutations and dN/dS number. The NJ tree was built with hierarchical clustering in R [113]. To address the difference between individual Peterhof strains by SNVs according to the S288C genome bedtools-intersect [114] with the -v option was used. SNV distribution in the genome was visualized with the ggplot2 package for R [115].

Copy number variation was estimated with the mrCaNaVaR pipeline v0.51 [107]. Subsequent analysis was performed with R v3.2 [113]. 1kb windows with normalized copy number above 1.8 were considered as amplified while those with copy number below 0.3 were considered as deleted. These windows were merged to calculate length of amplified or deleted regions and intersected with reference genome annotation to produce lists of presumably amplified or missing genes. The resulting gene lists were analyzed with YeastMine [116].

Genome tracks for nucleotide variation and genome assemblies visualized with UCSC Genome Browser [117] and GARfield are available at http://genome.ucsc.edu/cgi-bin/hgHubConnect#publicHubs and at http://garfield.dobzhanskycenter.org/cgi-bin/hgHubConnect, respectively.

Conservative chromosome regions were extracted from the 15V-P4, YS9 and RedStar assemblies with lastz [118] with default settings and manually curated. The YS9 and RedStar assemblies were downloaded from the *Saccharomyces* genome database. The corresponding sequences from the other strains were reported in [8] and downloaded at https://github.com/daskelly/yeast100genomes/. Multiple alignment of these regions from 95 or 97 strains was performed with MAFFT v7.182 [119, 120] in fftnsi mode. Neighbor-joining tree was also constructed with MAFFT.

For the ORF-based tree, ORF sets for different strains were downloaded from the *Saccharomyces* Genome Database. MAKER2 [32] was used to collect 15V-P4 ORFs, and in-house scripts were used to match them to the known reference genes, to intersect ORF sets and to distribute them into separate files, one for the each gene. Multiple alignment of common 807 ORFs was performed with MAFFT v7.182 [119, 120] in E-INS-i mode. Poorly aligned segments were filtered out with Gblocks v0.91b [121, 122] with a minimum block length equaling 6 bases and only positions where 50% or more of the sequences had a gap treated as a gap position. Maximum likelihood tree was constructed with RAxML v7.2.8 using rapid bootstrap analysis (-f a option) [123, 124].

Phylogenetic trees were visualized with the ape package for R [113] and with Figtree v1.4.0 [125].

Population structure analysis was performed with STRUCTURE v2.3.4 [35, 36]. For this, raw reads for the 15V-P4 genome were aligned to the reference S288C genome with BWA v0.6.1-r104 [126] in samse mode. SNV calling was performed with freebayes v1.0.2-6-g3ce827d [127] in haploid mode; SNVs with quality below 10 were filtered out. After that, these data were added to the data for 24,360 positions from the 100-genomes project [8] downloaded from https://github.com/daskelly/yeast100genomes/ with vcftools [111] and distributed into 10 equally spaced minor allele frequency bins with plink v1.9 [128, 129]. Then, 121 positions were randomly extracted from each of the bins 3 times and merged to create 3 datasets with equal representation of each frequency bin. These datasets were recoded with PGDSpider v2.1.0.0 [130] and used as input to STRUCTURE. STRUCTURE was run for 1,000,000 iterations with 200,000 burnin period for 6 clusters. The results were united with CLUMPP v1.1.2 [131] and visualized with R [113].

Custom scripts used for data analysis are available at https://github.com/drozdovapb/code_chunks/tree/master/Peterhof_strains_seq and https://github.com/drozdovapb/myBedGtfGffVcfTools.

### Plasmids

Plasmids YGPM27n09, YGPM11l14 and YGPM11e21 from the The Yeast Genomic Tiling Collection [132] were used to test complementation of the Gal- phenotype. Multicopy *LEU2* vector YEp351 [133] was used as a control.

pRS316-MSN4 was constructed by subcloning the 3.2 kb BamHI-EcoRI fragment from YGPM14i02 (The Yeast Genomic Tiling Collection; [132]) into the same sites of pRS316 [77]. pRS316-PHA2P, pRS316-pha2P-A10 and pRS316-pha2P-24 were constructed by cloning PCR products amplified with PHA2-F-BamHI and PHA2-R-BamHI (S8 Table) into the BamHI site of pRS316 [77]. *PHA2P* and *pha2P-24* were amplified using genomic DNA of 25-25-2V-P3982 as template (*pha2P-24* contains two PCR-induced missense mutations, N300S and F325S); *pha2P-A10* was amplified from 33G-D373 genomic DNA. The inserts were verified by sequencing (see S8 Table for information on the primers used); the same primers were used for sequencing of *PHA2* genomic allele. Sanger sequencing data were analyzed with UGENE [108].

### Phenotypic approaches

Standard yeast media [134] with minor modifications were used.

Yeast transformation was carried out according to the standard protocol [135] with modifications.

To test yeast abilities to grow in selective conditions, cells were suspended in water to equal OD_595_ and spotted on solid media in 5- or 10-fold serial dilutions.

To test cell aggregation, strains were inoculated in liquid YEPD medium and grown overnight at 26°C until reaching the stationary phase. Then the cultures were diluted tenfold with fresh media and grown for additional 4 hours. Aliquots were placed on microscopic slides and photographed (5 fields of view, Zeiss Primostar microscope, 400x magnification).

## Supporting Information

S1 Fig Pedigree of the strains. Names of strains with sequenced genomes are shown on yellow background. The number of generations is counted as the number of meiotic events between two strains. *MAT* **a** strains are depicted left-budded and *MAT* are right-budded. Diploids are unbudded. Curved arrow indicates self-fertilization. Dashed arrows indicate genetic manipulations without crossing. 25-25-2V-P3982 (the full name of the strain is 25-25-dU8-132-L28-2V-P3982) is an auxotrophic and suppressor mutant derivative of 2V-P3982 [28, 29]. 6P-33G-D373 is a 33G-D373 derivative in which *SUP35* is replaced with its homolog from *Pichia methanolica* [25].
(PDF)

S2 Fig Phylogenetic relation of 15V-P4 to other *S. cerevisiae* strains. (A) Population structure of 101 strains including 15V-P4 assessed with three sets of 1210 variable positions with roughly uniform minor allele frequency distribution. Populations or groups of similar populations are framed. 15V-P4 and S288C are highlighted in red. (B) Neighbor joining phylogenetic tree of 97 strains including 15V-P4, RedStar and YS9 inferred from alignment of conservative chromosome regions. Bakery strains (RedStar and YS9) are highlighted in violet, 15V-P4 and S288C are highlighted in red.
(PDF)

S3 Fig Coverage of *Saccharomyces sensu stricto* genomes with short reads for 15V-P4 does not reveal introgression from any of the closely related species. Short reads for the 15V-P4 genome were aligned to concatenated genomes of *S. sensu stricto* species with Bowtie2. S288C and YJM248 were used as a negative and positive controls for introgression, respectively. *Port*, *S. kudriavzevii* ZP 591. *Sbay*, *S. bayanus var. uvarum* CBS 7001. *Scer*, *S. cerevisiae* S288C. *Skud*, *S. kudriavzevii* IFO1802^T^. *Smik*, *S. mikatae* IFO1815^T^. *Spar*, *S. paradoxus* CBS432. *Seub*, *S. eubayanus* FM1318. *Sarb*, *S. arboricolus* H-6.
(PDF)

S4 Fig Genome coverage across reference for euploid strains. (A) 1B, (B) 74. Dashed lines signify chromosome borders.
(PDF)

S5 Fig Neighbour joining (NJ) clustering of the PGC strains and S288C based on number of pairwise SNVs. Shown in right are numbers of SNVs in comparison to S288C (highlighted in different shades of green with color intensity proportional to the number of SNVs) or to 15V-P4 (similarly highlighted in shades of purple).
(PDF)

S6 Fig Phenylalanine auxotrophy mutation *pheA10* is allelic to *PHA2*. (A) Short read alignment. (B) Sanger resequencing. Red frame, TAA nonsense mutation appearing at codon 161. (C) 33G-D373 plated on selective media immediately after transformation with low copy number plasmids bearing indicated *PHA2* alleles. Vector, pRS316. (D) Asp^+^ and Asp^-^ variants of 6P-33G-D373 spotted for nonsense suppression and copper resistance. Series of 5-fold dilutions are shown.
(PDF)

S7 Fig Nonsense mutation in *MSN4* does not contribute to thermosensitivity. Introduction of a centromeric plasmid with the wild type *MSN4* allele does not influence thermotolerance in 74-D694 ([*psi*^-^]) and P-74-D694 ([*PSI*^+^]). Series of 5-fold dilutions are shown. Vector, pRS316.
(PDF)

S8 Fig *GAL10*^C287T^ mutation in the 1B strain may be responsible for the Gal- phenotype. (A) SNVs in the *GAL* locus compared to S288C. Upper character, reference nucleotide; lower character, variant nucleotide. Nucleotides of the Watson strand are indicated. C287T substitution in *GAL10* of 1B is highlighted in blue circle. (B) The complete *GAL* locus or its *GAL10*-containing fragment but not *GAL1* alone compensates for 1B inability to grow on galactose-containing medium. 1B was transformed with multicopy plasmids containing the complete *GAL* locus (*GAL7*+*GAL10*+*GAL1*) or its fragments containing either only *GAL1* or *GAL7*+*GAL10*. Shown are series of 10-fold dilutions spotted on synthetic media lacking leucine with glucose or galactose/raffinose as a carbon source. Vector, YEp351. (C) Alignment of conservative part of UDP-galactose-4-epimerase homologs (Gal10 from *S. cerevisiae* S288C and 1B strains and GalE proteins from other species). In blue frame, Ala96Val substitution in 1B. In red frame, 94Val in human GALE.
(PDF)

S1 Table Systematic names of genes used to infer the ORF-based phylogenetic tree. (XLS)

S2 Table Summary of variable positions in the *SUCX* genes. Positions are indicated according to S288C *SUC2* sequence. Variants are called according to short read alignment for sequenced PGC strains and to ungapped multiple alignment for known *SUC* genes (NCBI accession numbers are given in parentheses).
(XLS)

S3 Table Summary of BLAST analysis for introgressed regions. Shown are results of BLAST search (output format 6) in the 15V-P4 genome and in the YJM248 genome.
(XLS)

S4 Table Genomic regions annotated as amplified or deleted in each of the genomes. (XLS)

S5 Table Lists of genes annotated as amplified or deleted in each of the genomes and their functional characteristics. Genes that are classified as amplified because they have close paralogs or similar sequences somewhere else in the genome are highlighted in beige, those residing in amplified chromosomes in gray, common deleted genes in orange, and known genotypic changes in green.
(XLS)

S6 Table Number of SNVs and short indels in each of the genomes analyzed. (PDF)

S7 Table List of genes with stop codons gained or lost in the strains analyzed. Light green, known genotype.
(XLS)

S8 Table Primers used in this work. (XLS)

S1 Appendix Genetic variations previously described in 25-25, 1B, 74, and 6P-33G compared to S288C and 15V-P4. (PDF)
