# Supplementary material for: Genome Sequencing and Comparative Analysis of Saccharomyces cerevisiae Strains of the Peterhof Genetic Collection
Source: PLoS One. 2016 May 6;11(5):e0154722. doi: 10.1371/journal.pone.0154722 (PMC4859572; doi:10.1371/journal.pone.0154722)
Supplement: S1 Fig — Names of strains with sequenced genomes are shown on yellow background. The number of generations is counted as the number of meiotic events between two strains. MAT a strains are depicted left-budded and MAT are right-budded. Diploids are unbudded. Curved arrow indicates self-fertilization. Dashed arrows indicate genetic manipulations without crossing. 25-25-2V-P3982 (the full name of the strain is 25-25-dU8-132-L28-2V-P3982) is an auxotrophic and suppressor mutant derivative of 2V-P3982 [28, 29]. 6P-33G-D373 is a 33G-D373 derivative in which SUP35 is replaced with its homolog from Pichia methanolica [25]. (PDF) [file pone.0154722.s001.pdf]

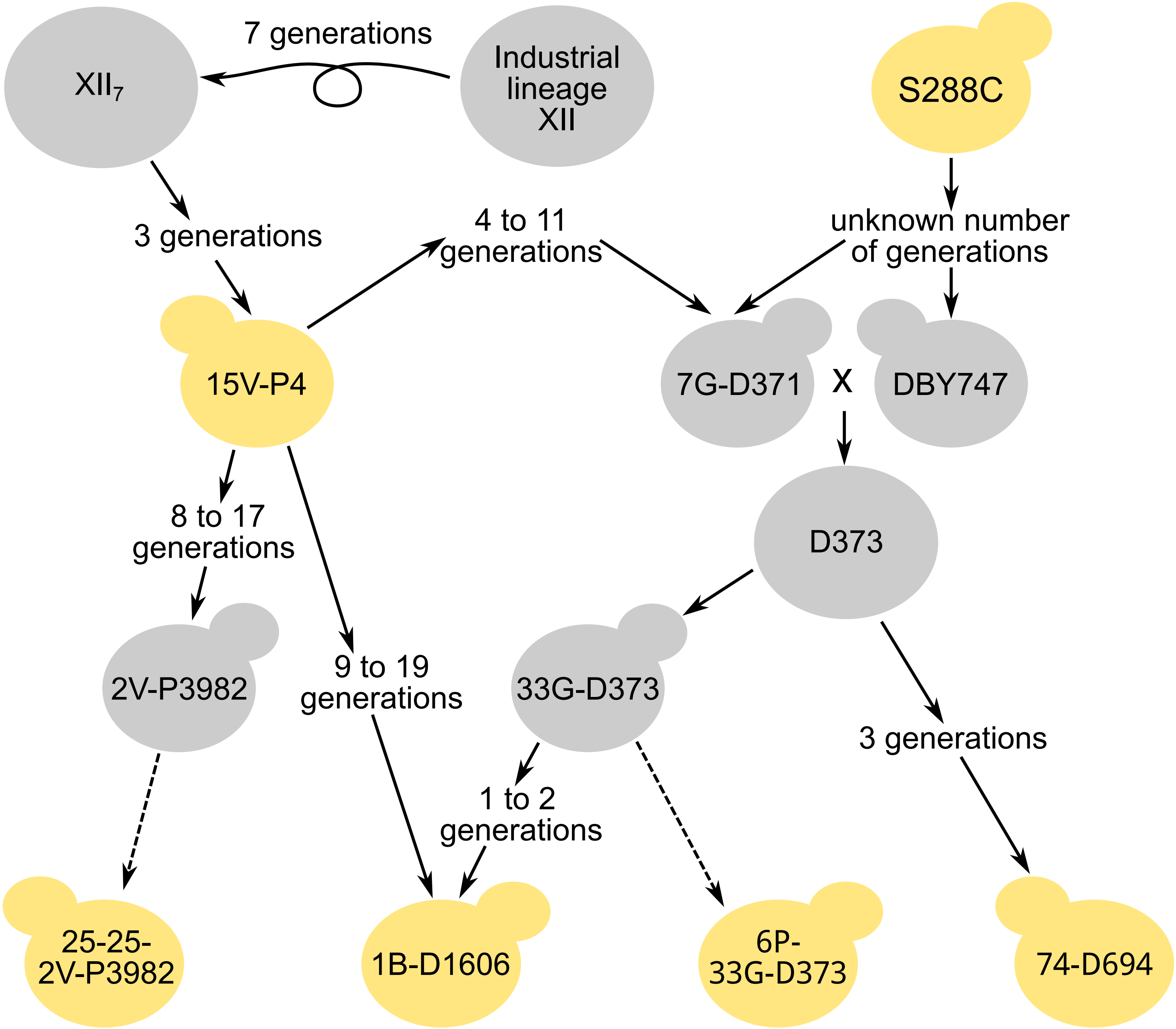

S1 Fig

Pedigree of the strains

Names of strains with sequenced genomes are shown on yellow background. The number of generations is counted as the number of meiotic events between two strains. *MAT***a** strains are depicted left-budded and *MATα* are right-budded. Diploids are unbudded. Curved arrow indicates self-fertilization. Dashed arrows indicate genetic manipulations without crossing. 25-25-2V-P3982 (the full name of the strain is 25-25-dU8-132-L28-2V-P3982) is an auxotrophic and suppressor mutant derivative of 2V-P3982 [23, 24]. 6P-33G-D373 is a 33G-D373 derivative in which *SUP35* is replaced with its homolog from *Pichia methanolica* [25].
