# Supplementary material for: Genome Sequencing and Comparative Analysis of Saccharomyces cerevisiae Strains of the Peterhof Genetic Collection
Source: PLoS One. 2016 May 6;11(5):e0154722. doi: 10.1371/journal.pone.0154722 (PMC4859572; doi:10.1371/journal.pone.0154722)

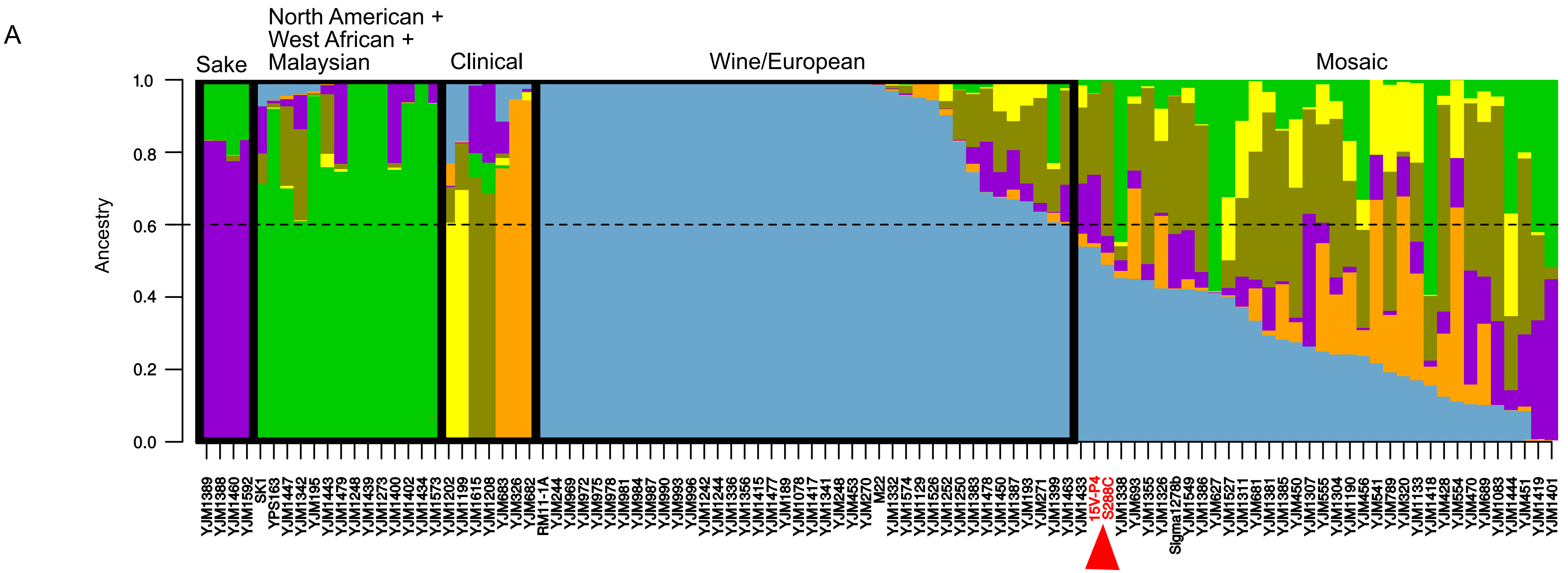

S2 Fig
Phylogenetic relation of 15V-P4 to other *S. cerevisiae* strains.
(A) Population structure of 101 strains including 15V-P4 assessed with three sets of 1210 variable positions with roughly uniform minor allele frequency distribution. Populations or groups of similar populations are framed. 15V-P4 and S288C are highlighted in red.
(B) Neighbor joining phylogenetic tree of 97 strains including 15V-P4, RedStar and YS9 inferred from alignment of conservative chromosome regions. Bakery strains (RedStar and YS9) are highlighted in violet, 15V-P4 and S288C are highlighted in red.

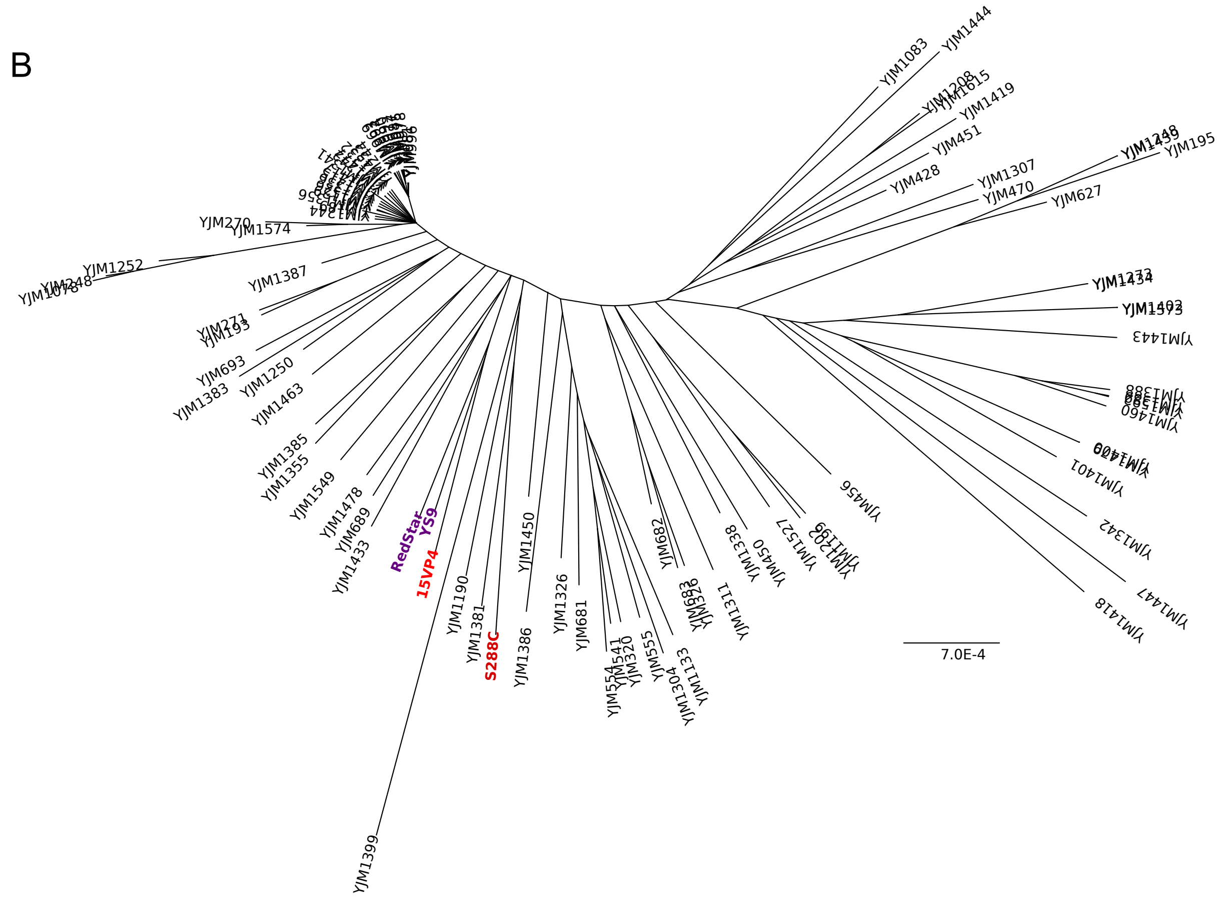

Supplement: S2 Fig — (A) Population structure of 101 strains including 15V-P4 assessed with three sets of 1210 variable positions with roughly uniform minor allele frequency distribution. Populations or groups of similar populations are framed. 15V-P4 and S288C are highlighted in red. (B) Neighbor joining phylogenetic tree of 97 strains including 15V-P4, RedStar and YS9 inferred from alignment of conservative chromosome regions. Bakery strains (RedStar and YS9) are highlighted in violet, 15V-P4 and S288C are highlighted in red. (PDF) [file pone.0154722.s002.pdf]
