# Supplementary material for: Genome Sequencing and Comparative Analysis of Saccharomyces cerevisiae Strains of the Peterhof Genetic Collection
Source: PLoS One. 2016 May 6;11(5):e0154722. doi: 10.1371/journal.pone.0154722 (PMC4859572; doi:10.1371/journal.pone.0154722)
Supplement: S3 Fig — Short reads for the 15V-P4 genome were aligned to concatenated genomes of S. sensu stricto species with Bowtie2. S288C and YJM248 were used as a negative and positive controls for introgression, respectively. Port, S. kudriavzevii ZP 591. Sbay, S. bayanus var. uvarum CBS 7001. Scer, S. cerevisiae S288C. Skud, S. kudriavzevii IFO1802T. Smik, S. mikatae IFO1815T. Spar, S. paradoxus CBS432. Seub, S. eubayanus FM1318. Sarb, S. arboricolus H-6. (PDF) [file pone.0154722.s003.pdf]

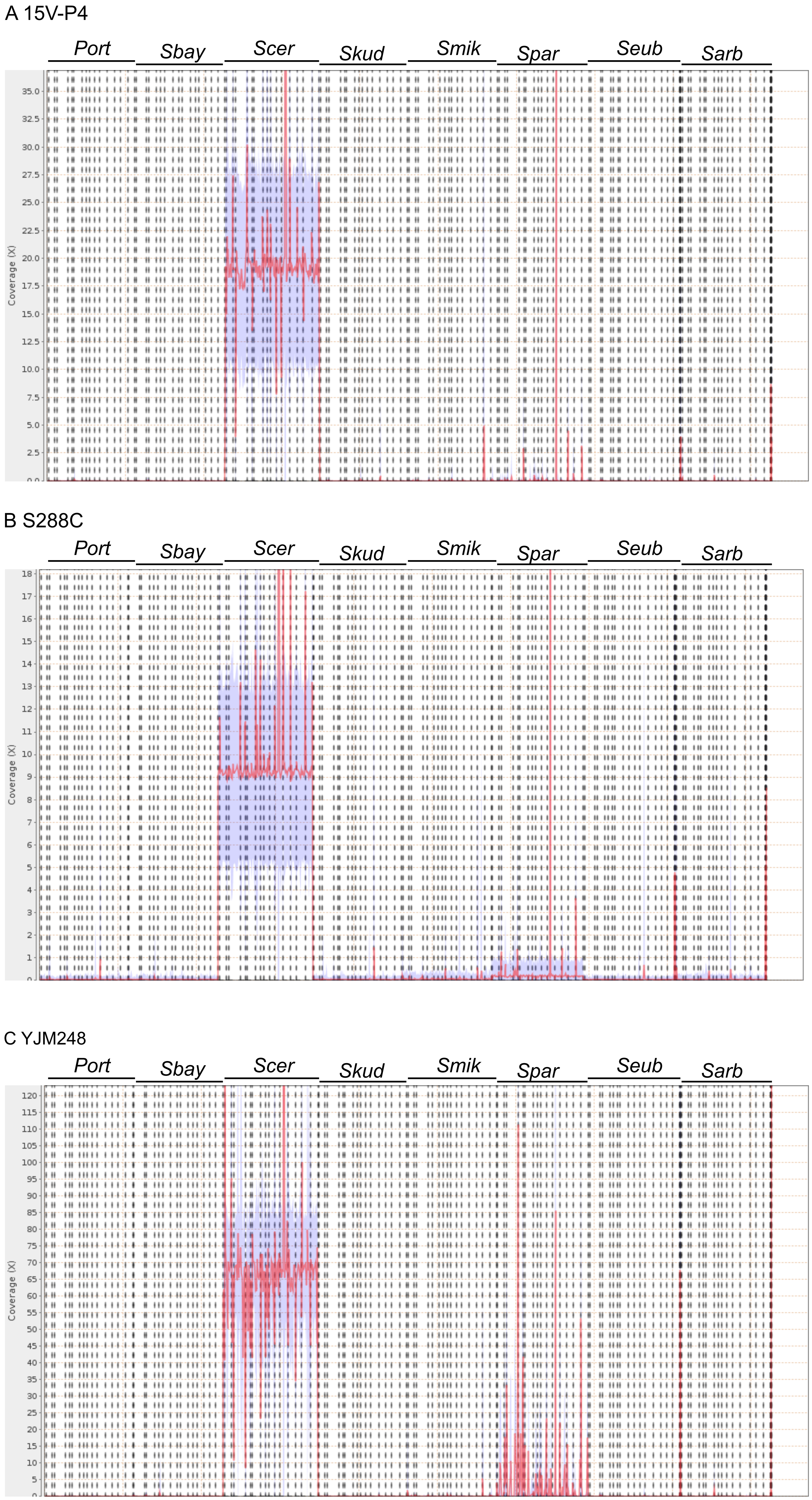

Figure S3. Coverage of *Saccharomyces sensu stricto* genomes with short reads for 15V-P4 does not reveal introgression from any of the closely related species. Short reads for the 15V-P4 genome were aligned to concatenated genomes of *S. sensu stricto* species with bowtie2. S288C and YJM248 were used as a negative and positive controls for introgression, respectively.

Port, *S. kudriavzevii* ZP 591
Sbay, *S. bayanus var. uvarum* CBS 7001
Scer, *S. cerevisiae* S288C
Skud, *S. kudriavzevii* IFO1802^T
Smik, *S. mikatae* IFO1815^T
Spar, *S. paradoxus* CBS432
Seub, *S. eubayanus* FM1318
Sarb, *S. arboricolus* H-6
