# Supplementary material for: Genome Sequencing and Comparative Analysis of Saccharomyces cerevisiae Strains of the Peterhof Genetic Collection
Source: PLoS One. 2016 May 6;11(5):e0154722. doi: 10.1371/journal.pone.0154722 (PMC4859572; doi:10.1371/journal.pone.0154722)
Supplement: S4 Fig — (A) 1B, (B) 74. Dashed lines signify chromosome borders. (PDF) [file pone.0154722.s004.pdf]

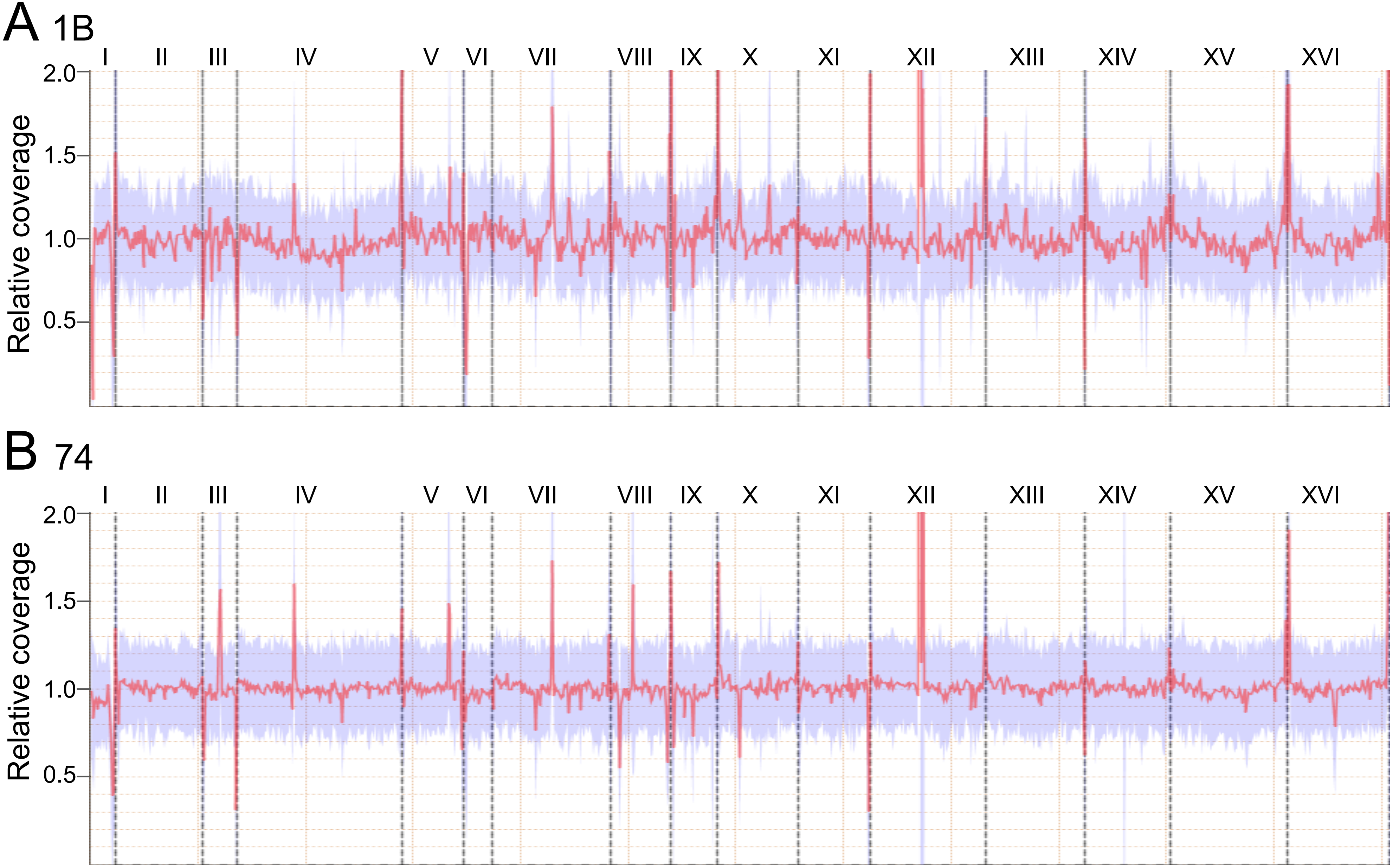

S4 Fig

Genome coverage across reference for euploid strains. (A) 1B, (B) 74. Dashed lines signify chromosome borders.
