# Supplementary material for: Genome Sequencing and Comparative Analysis of Saccharomyces cerevisiae Strains of the Peterhof Genetic Collection
Source: PLoS One. 2016 May 6;11(5):e0154722. doi: 10.1371/journal.pone.0154722 (PMC4859572; doi:10.1371/journal.pone.0154722)
Supplement: S5 Fig — Shown in right are numbers of SNVs in comparison to S288C (highlighted in different shades of green with color intensity proportional to the number of SNVs) or to 15V-P4 (similarly highlighted in shades of purple). (PDF) [file pone.0154722.s005.pdf]

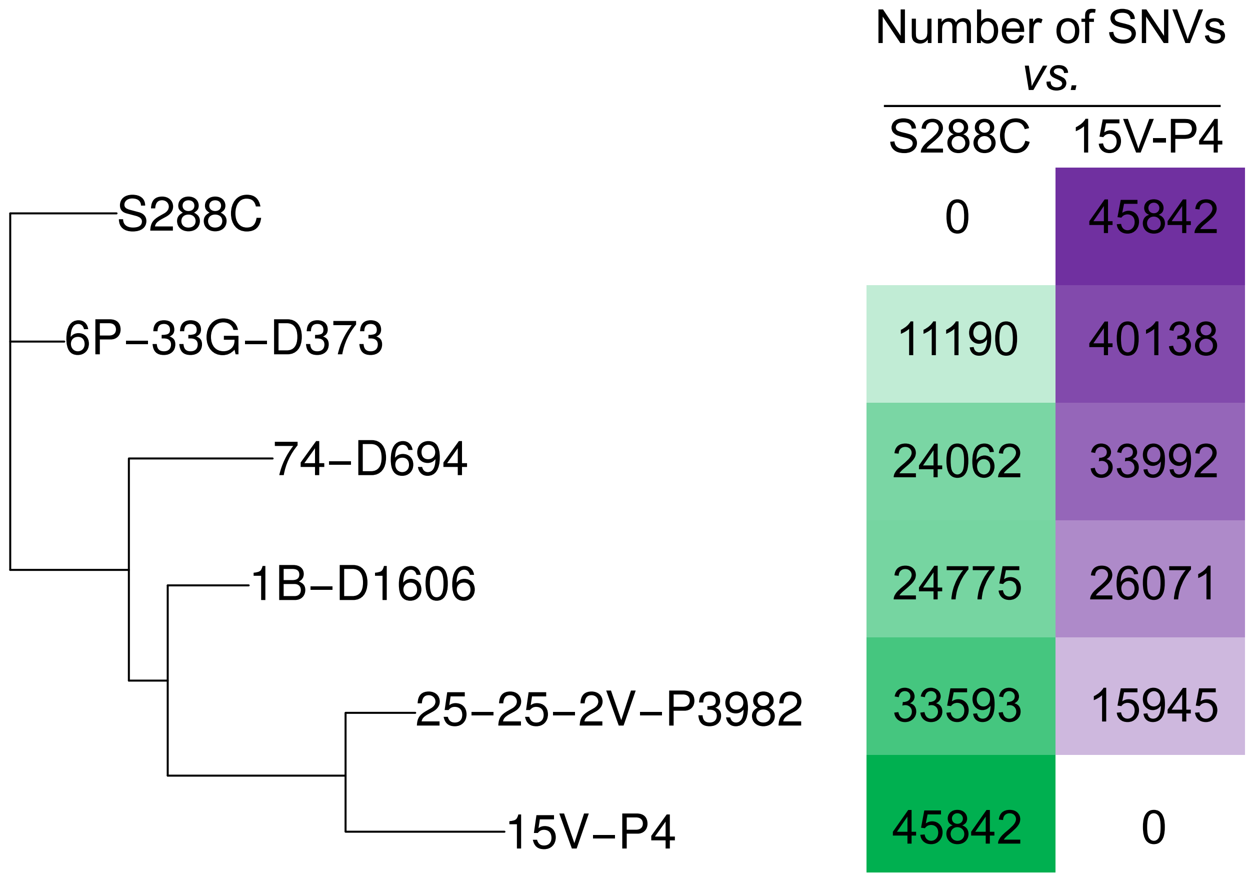


S5 Fig
**Neighbour joining clustering of the PGC strains and S288C based on number of pairwise SNVs**
Shown in right are numbers of SNVs in comparison to S288C (highlighted in different shades of green with color intensity proportional to the number of SNVs) or to 15V-P4 (similarly highlighted in shades of purple).
