# Supplementary material for: Genome Sequencing and Comparative Analysis of Saccharomyces cerevisiae Strains of the Peterhof Genetic Collection
Source: PLoS One. 2016 May 6;11(5):e0154722. doi: 10.1371/journal.pone.0154722 (PMC4859572; doi:10.1371/journal.pone.0154722)
Supplement: S6 Fig — (A) Short read alignment. (B) Sanger resequencing. Red frame, TAA nonsense mutation appearing at codon 161. (C) 33G-D373 plated on selective media immediately after transformation with low copy number plasmids bearing indicated PHA2 alleles. Vector, pRS316. (D) Asp+ and Asp- variants of 6P-33G-D373 spotted for nonsense suppression and copper resistance. Series of 5-fold dilutions are shown. (PDF) [file pone.0154722.s006.pdf]

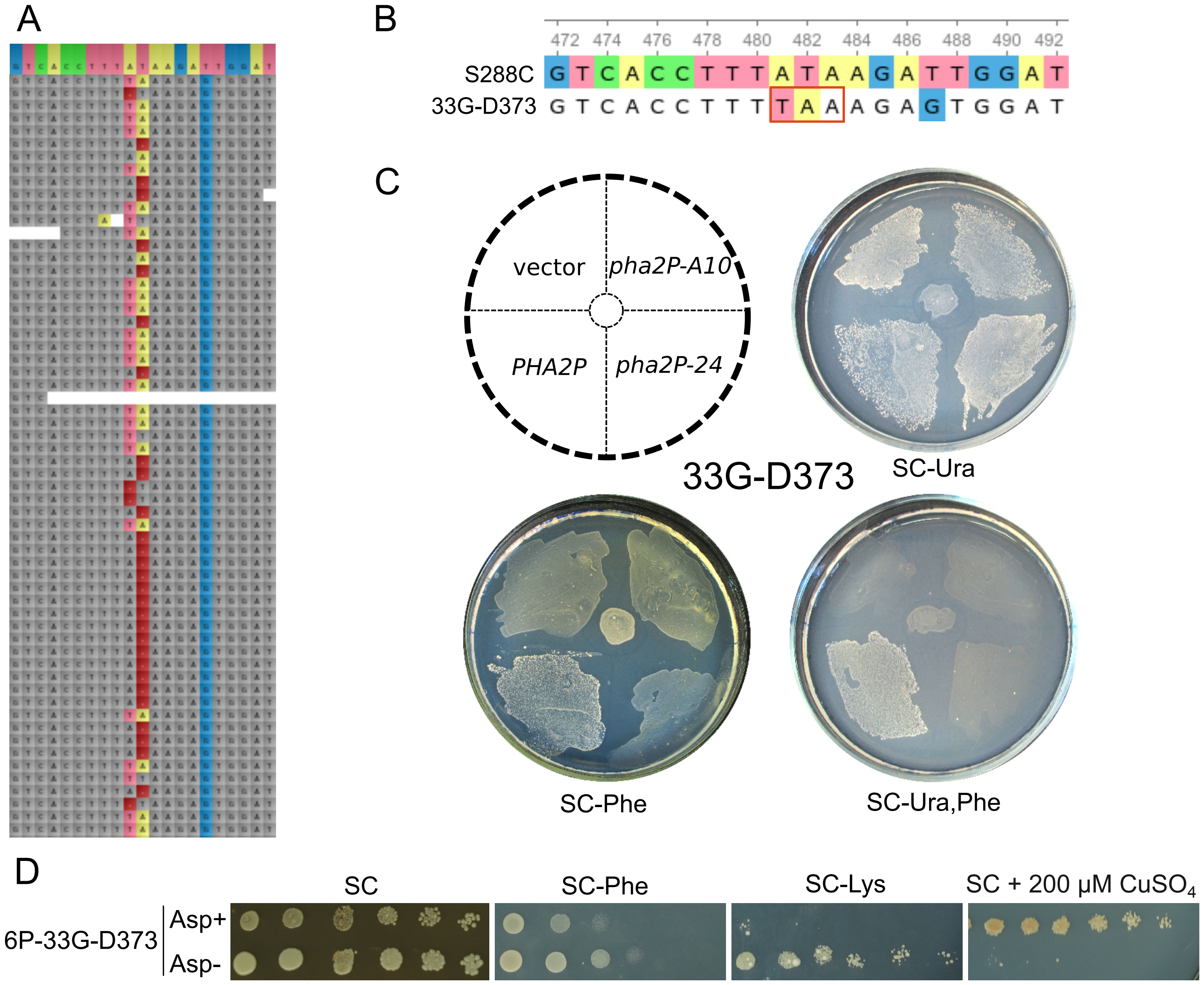

S6 Fig

Phenylalanine auxotrophy mutation *pheA10* is allelic to *PHA2*

(A) Short read alignment. (B) Sanger resequencing. Red frame, TAA nonsense mutation appearing at codon 161. (C) 33G-D373 plated on selective media immediately after transformation with low copy number plasmids bearing indicated *PHA2* alleles. Vector, pRS316. (D) Asp^+ and Asp^- variants of 6P-33G-D373 spotted for nonsense suppression and copper resistance. Series of 5-fold dilutions are shown.
