# Supplementary material for: Genome Sequencing and Comparative Analysis of Saccharomyces cerevisiae Strains of the Peterhof Genetic Collection
Source: PLoS One. 2016 May 6;11(5):e0154722. doi: 10.1371/journal.pone.0154722 (PMC4859572; doi:10.1371/journal.pone.0154722)
Supplement: S7 Fig — Introduction of a centromeric plasmid with the wild type MSN4 allele does not influence thermotolerance in 74-D694 ([psi-]) and P-74-D694 ([PSI+]). Series of 5-fold dilutions are shown. Vector, pRS316. (PDF) [file pone.0154722.s007.pdf]

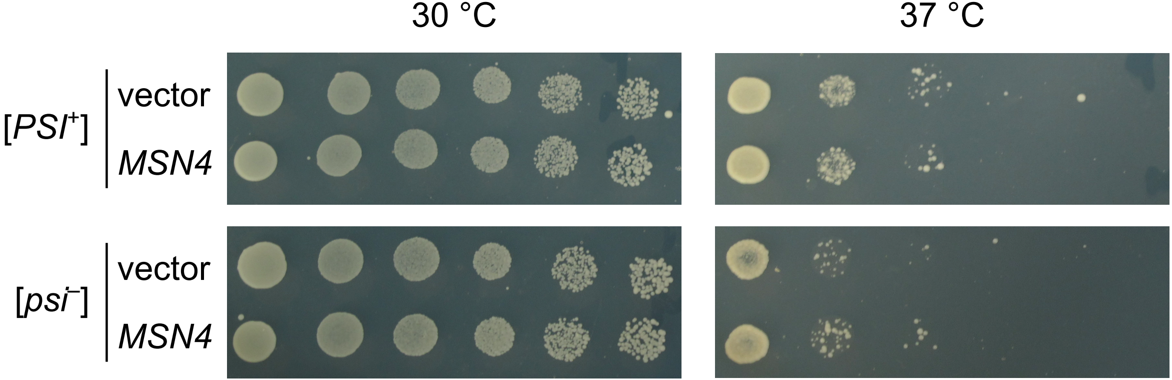

S7 Fig

Nonsense mutation in *MSN4* does not contribute to thermosensitivity

Introduction of a centromeric plasmid with the wild type *MSN4* allele does not influence thermotolerance in 74-D694 ([*psi*$^-$]) and P-74-D694 ([*PSI*$^+$]). Series of 5-fold dilutions are shown. Vector, pRS316.
