# Supplementary material for: Genome Sequencing and Comparative Analysis of Saccharomyces cerevisiae Strains of the Peterhof Genetic Collection
Source: PLoS One. 2016 May 6;11(5):e0154722. doi: 10.1371/journal.pone.0154722 (PMC4859572; doi:10.1371/journal.pone.0154722)
Supplement: S8 Fig — (A) SNVs in the GAL locus compared to S288C. Upper character, reference nucleotide; lower character, variant nucleotide. Nucleotides of the Watson strand are indicated. C287T substitution in GAL10 of 1B is highlighted in blue circle. (B) The complete GAL locus or its GAL10-containing fragment but not GAL1 alone compensates for 1B inability to grow on galactose-containing medium. 1B was transformed with multicopy plasmids containing the complete GAL locus (GAL7+GAL10+GAL1) or its fragments containing either only GAL1 or GAL7+GAL10. Shown are series of 10-fold dilutions spotted on synthetic media lacking leucine with glucose or galactose/raffinose as a carbon source. Vector, YEp351. (C) Alignment of conservative part of UDP-galactose-4-epimerase homologs (Gal10 from S. cerevisiae S288C and 1B strains and GalE proteins from other species). In blue frame, Ala96Val substitution in 1B. In red frame, 94Val in human GALE. (PDF) [file pone.0154722.s008.pdf]

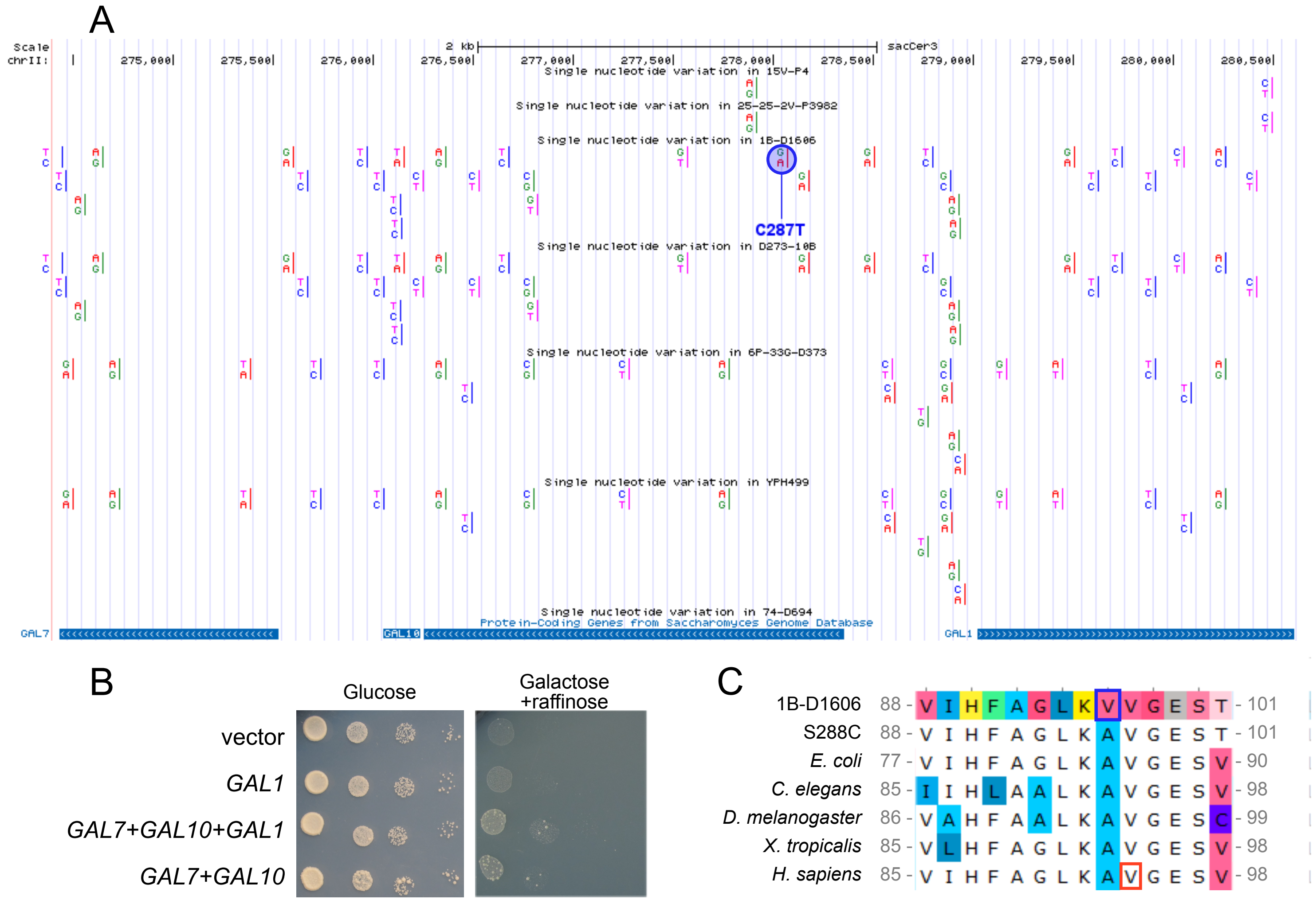

S8 Fig

***GAL10*^{C287T} mutation in the 1B-D1606 strain may be responsible for the Gal- phenotype**

(A) SNVs in the *GAL* locus compared to S288C. Upper character, reference nucleotide; lower character, variant nucleotide. Nucleotides of the Watson strand are indicated. C287T substitution in GAL10 of 1B is highlighted in blue circle.

(B) The complete *GAL* locus or its *GAL10*-containing fragment but not *GAL1* alone compensates for 1B inability to grow on galactose-containing medium. 1B was transformed with multicopy plasmids containing the complete *GAL* locus (*GAL7+GAL10+GAL1*) or its fragments containing either only *GAL1* or *GAL7+GAL10*. Shown are series of 10-fold dilutions spotted on synthetic media lacking leucine with glucose or galactose/raffinose as a carbon source. Vector, YEp351.

(C) Alignment of conservative part of UDP-galactose-4-epimerase homologs (Gal10 from *S. cerevisiae* S288C and 1B strains and GalE proteins from other species). In blue frame, Ala96Val substitution in 1B. In red frame, 94Val in human GALE.
