# Supplementary material for: Genome Sequencing and Comparative Analysis of Saccharomyces cerevisiae Strains of the Peterhof Genetic Collection
Source: PLoS One. 2016 May 6;11(5):e0154722. doi: 10.1371/journal.pone.0154722 (PMC4859572; doi:10.1371/journal.pone.0154722)
Supplement: S6 Table — (PDF) [file pone.0154722.s014.pdf]

S6 Table. Number of SNVs and short indels in each of the genomes analyzed.

Strain	SNVs	Indels	100% supported indels	Sequencing technology
15V-P4	45,842	4,728	1,934	IonTorrent
25-25	33,593	4,297	1,518	IonTorrent
1B	24,775	5,270	1,234	IonTorrent
74	24,062	1,125	1,106	Illumina
6P-33G	11,190	6,392	650	IonTorrent
