# Supplementary material for: Genome Sequencing and Comparative Analysis of Saccharomyces cerevisiae Strains of the Peterhof Genetic Collection
Source: PLoS One. 2016 May 6;11(5):e0154722. doi: 10.1371/journal.pone.0154722 (PMC4859572; doi:10.1371/journal.pone.0154722)
Supplement: S1 Appendix — (PDF) [file pone.0154722.s017.pdf]

S1 Appendix. Genetic variations previously described in 25-25-2V-P3982, 1B-D1606, 74-D694, and 6P-33G-D373 compared to S288C and 15V-P4.

Gene	15V-P4 (PGC substitutions)	25-25-2V-P3982	1B-D1606	74-D694	6P-33G-D373	References
SUP35	C198T, A326G, G485A, T507A, C556G, C617A, T654C, C673G, C1167T, C1347T	PGC substitutions + C1133T (*sup35-25*)	—	—	*SUP35* from *Pichia methanolica*	[27, 28, 25]
SUP45	-234ΔT, -165insT, G-34A, A69G, G519T, T687C, T702A, G927A, T1008C	PGC substitutions + G1199T (*sup45-400*)	PGC substitutions, excluding -165insT	—	—	[136, 15, 23]
ADE1	T102C, T264C, A456G	PGC substitutions + G732A (*ade1-14*)	PGC substitutions + G732A (*ade1-14*)	PGC substitutions + G732A (*ade1-14*)	—	[52-54]
ADE2	T-19C, A27G, A66T, A301G, T372C, G1617A	—	PGC substitutions	PGC substitutions	PGC substitutions + T422A, G423A, C1517T (*ade2-144,717* = *ade2-144,791*)*	[55, 56], and this work
HIS3	—	—	—	Deletion (-206 to +171) (*his3Δ200*)	—	[57]
HIS7	C-18T, G-2A, T900C, C1212T	PGC substitutions + A229T (*his7-1*)	PGC substitutions + A229T (*his7-1*)	T-81C, A-27G, C-18T, G-2A, T138C, T534C, T1134C	PGC substitutions + A229T (*his7-1*)	[58, 59]
LEU2	T381C, C450T, T783C, C864T, T870C, G969A	PGC substitutions + G748A** (*leu2-B2*)	249insG, 792insG (*leu2-3,112*)	249insG, 792insG (*leu2-3,112*)	—	This work, [60]
LYS2	C322A, C498T, A1074G, A1196T, C1209T, T1608G, G2040C, T2925C, A3054G, T3318C, T3345A, G3390A, G3550A, A3777G, G3873A, C4111T, A4161G	PGC substitutions + G3465A*** (*lys2-87 = L28*)	PGC substitutions	PGC substitutions	PGC substitutions	[52] and this work
LYS9	A303G, C1116T, T1170G	PGC substitutions	PGC substitutions + T605A (*lys9-A21*)	A-49G, A6G, G9A, T207C, A303G, C486T, G717C, G819A	PGC substitutions + T605A (*lys9-A21*)	[58, 59]
PHA2	T257C, A306G, G394A, G447C, T482A, T487G, T716C, C735T, C745G, C891T	PGC substitutions	—	—	PGC substitutions + A481T**** (*pheA10*), T588C	[61], unpublished data, and this work
THR4	T335G	PGC substitution + A1180T*** (*thr4-B15*)	—	PGC substitution	—	[52] and this work
TRP1	C403A	PGC substitution	C403T (*trp1-289*)	C403T (*trp1-289*)	C403T (*trp1-289*)	[62, 59]
URA3	—	Deletion -188 to +93 (*dU8*)	Ty insertion at 121 (*ura3-52*)	Ty insertion at 121 (*ura3-52*)	Ty insertion at 121 (*ura3-52*)	Unpublished data, [63]

* *ade2-144,717* was initially described as *ade2-144-791* and then renamed.
** only the phenotype and the corresponding gene but not the mutation type was known.
*** only the stop codon type in the gene was known.
**** only the phenotype and the stop codon type but not the corresponding gene was known.

Nucleotide positions in 5’ UTR are preceded with the minus sign while those in 3’ UTR with the plus sign; numbers indicate distance from the beginning or the end of the ORFs, respectively.

The nonsense (or changing nonsense type), missense, samesense mutations, as well as insertions/deletions in ORFs and mutations in intergenic regions are highlighted in the respective color. Names of previously described mutations are given in parentheses following the corresponding variation and colored similarly.

Commentary

SUP35

The first data about differences between Peterhof and reference *SUP35* alleles were obtained by Volkov *et al.* [27]. The PGC allele, *SUP35P*, was shown to differ in 10 nucleotide positions, 6 of which led to missense mutations. In the current work we confirmed the presence of all the described substitutions in the pure Peterhof strains, but not in those of a hybrid origin, suggesting that 1B-D1606 and 74-D694 had inherited S288C-derived *SUP35* allele. Importantly, none of the amino acid substitutions are localized in the conservative C-terminal domain of Sup35, which is necessary and sufficient for cell viability and robust termination of translation (see [137]). Only one substitution is located in the N domain, which is responsible for prion and amyloid properties of Sup35 (see [137]). Five substitutions reside in the M domain, the exact function of which is still unclear, but it was shown to affect propagation of the [*PSI*$^+$] prion [138], so it would be interesting to find out whether PGC substitutions in *SUP35P* affect properties of [*PSI*$^+$].

The *sup35-25* allele was selected as a nonsense suppressor mutation in the 25-25-2V-P3982 closest ancestor, dU8-132-L28-2V-P3982 [28]. Sequencing revealed T378I substitution in the C domain, which distinguished *sup35-25* from the wild-type Peterhof allele [28]. Genome sequencing of 25-25-2V-P3982 successfully identified *sup35-25* substitution confirming previous findings.

We did not detect *S. cerevisiae SUP35* in 6P-33G-D373, consistent with the native ORF in this strain being substituted for the *PmSUP35* allele cloned from the *Pichia methanolica* genome [25]. Exonerate search in the 6P-33G-D373 genome for the Sup35 N domain sequence from *Pichia methanolica* [139] did return this sequence.

SUP45

For the first time the PGC-derived allele of *SUP45* was cloned from the 7B-D244 strain [140], which contained the *sup1-ts36* mutation. This allele became one of the two first sequenced *SUP45* alleles from *S. cerevisiae* [136]. Sequencing and comparison of the *sup1-ts36* and S288C-derived *SUP45* alleles revealed variations in the promoter as well as six substitutions in the ORF [136]. Only one substitution led to a missense mutation which was proved to be responsible for the *sup1-ts36* phenotype by complementation analysis in fragment exchange experiment. All the other nucleotide variations in the ORF were synonymous and were suggested to derive from Peterhof ancestors of 7B-D244 [136]. Later, the same five samesense substitutions in *SUP45* ORF were found in the allele from 1B-D1606; however, the upstream sequence of the gene was not analyzed [15]. In this work we confirmed that all the substitutions in the *SUP45* ORF are present in ancestor strain, 15V-P4, as well as in 25-25-2V-P3982 and 1B-D1606, which proves that this substitutions are indeed characteristic for PGC. Furthermore, we found the same variations, as described by Breining and Piepersberg [136], in the *SUP45* promoter sequences. Whether these variations affect *SUP45* expression in PGC strains might be a subject for further investigations.

The observation that addition of a wild type *SUP45* copy changed nonsense suppression efficiency in 25-25-2V-P3982 led to discovery of the *sup45-400* mutation in this strain ([23] and N. Rovinsky, unpublished data). However, the mechanism by which *sup45-400* affects nonsense suppression remains elusive. In this work, we found the same nucleotide change as described.

ADE1

The *ade1-14* mutation was isolated in p14-15V-P4 ("p" indicating "pink" color phenotype) as an adenine auxotrophy accompanied by red pigment accumulation which was shown to be allelic to *ade1* mutations [17]. After demonstration of *ade1-14* usefulness in nonsense suppression study, while disclosing the nature of the mutation as an TGA nonsense [49], the allele became widely used throughout the world primarily as a reporter for the [*PSI*$^+$] nonsense-suppressor phenotype.

Two independent studies presented sequencing data for *ade1-14*. Bertram *et al.* reported Trp244[TGA] substitution in the allele isolated from IS31Δ7b/1c strain [53]. Unfortunately, we could not track the origin of the strain which came as a source of the allele in the work of Bertram *et al.*, as we faced the common problem that the researchers tend to rename the strains with no respect to their origin. Sequencing of the same *ade1-14* allele from 74-D694 by Nakayashiki *et al*. [54] found a missense mutation at codon 185 in addition to the previously described nonsense codon. Our sequencing data for three strains containing *ade1-14*, including 74-D694, confirmed the TGG to TGA change at codon 244 as well as three 15V-P4-derived synonymous substitutions, but no missense mutations were detected. Thus we conclude that the missense mutation described by Nakayashiki *et al.* [54] emerged in their laboratory and is not characteristic to the *bona fide ade1-14* allele.

ADE2

ade2-144 was found among UV- or X-ray-induced adenine auxotrophy mutations obtained in 15V-P4 (p144-15V-P4). It was shown to be suppressible by dominant suppressors, *i.e.*, to be a nonsense mutation [55], but the stop codon type has never been reliably identified, although it was hypothesized to be a TAG [141]. *ADE2* gene has been extensively used to study interallelic and intragenic complementation [56, 142, 143], and a number of double mutants was obtained. One of them, *ade2-144,791* [56], which later had been occasionally renamed as *ade2-144,717* (unpublished), was inherited by 33G-D373 [65]. None of the studies we are aware of addressed the nature of *ade2-791* mutation, but it was known to be located downstream of *ade2-144* and appeared not to be a nonsense mutation [56]. In this work we found one missense and four synonymous substitutions in the *ADE2* allele of PGC compared to reference. Genome sequencing of the 6P-33G-D373 strain showed that the *ade2-144,717* allele, compared to *ADE2* from 15V-P4, did contain P506L missense downstream the TAA nonsense mutation. Interestingly, *ade2-144* TAA nonsense is a two-nucleotide substitution. The fact that early studies suggested *ade2-144* to be an amber mutation leads to a speculation that only T422A substitution could have been present initially, and G423A (highlighted in pale red in the table) might have been acquired later during subsequent breeding and selection, changing the stop codon from TAG to TAA.

Surprisingly, 25-25-2V-P3982 bears S288C-like *ADE2* allele, which contradicts the presumed pure Peterhof origin of the strain, as well as SNVs distribution analysis does.

HIS3

74-D694 is the only strain analyzed that exploits the *his3* selectable marker, as it contains the null allele *his3Δ200* inherited from D373 (see S1 Fig). Copy number variation analysis confirmed that *HIS3* was absent in 74-D694 but not in other strains. Manual inspection of the short read alignment allowed us to determine the borders of the deleted region which corresponded well to the known coordinates of the deletion [57]. *HIS3* allele in four other strains is identical to the reference, representing a rare example of absence of PGC substitutions in the gene.

HIS7

his7-1 was identified as an ochre nonsense mutation by Simarov *et al*. (see [58]). Sequencing of the allele from 1B-D1606 showed AAA to TAA substitution at codon 77 [59]. We confirmed the presence of this nonsense mutation and additionally found two 15V-P4-derived synonymous substitutions in all the *his7-1* strains. Interestingly, *HIS7* from 74-D694 contains three SNVs different from those of PGC but shared by some other *S. cerevisiae* strains (*e.g.*, D273-10B) suggesting that *HIS7* in 74-D694 is of a non-PGC origin.

LEU2

Present in the 25-25-2V-P3982 genome, *leu2-1* is a non-suppressible mutation of unknown nature (L. Murashko, unpublished data). We were able to track the origin of this allele: it was isolated upon UV irradiation of 4B-2B-(4-10), a haploid strain of the Peterhof origin, obtained with two meioses of a tetraploid strain. The resulting leucine auxotroph was designated B2-4B-2B-(4-10) [17]. Analogously to other alleles of similar origin, the allele should be named *leu2-B2*; instead, it became erroneously referred to as *leu2-1* [27, 52]. It has also been designated *leu2-01* [12] and *leu2-1A* [61] (probably to avoid confusion with the original *leu2-1* mutation obtained independently [144]). We thus suggest *leu2-B2* as a designation for this mutation.

We found six synonymous substitutions in both 15V-P4 and 25-25-2V-P3982 genomes, and only in the latter one additional missense mutation (D250N), which we conclude to be responsible for the leucine auxotrophy and to correspond to the *leu2-B2* allele.

leu2-3,112 is a double frameshift mutation obtained via recombination between *leu2-3* and *leu2-112* alleles [145], which were shown to be single-nucleotide G insertions in positions 249 and 792 of *LEU2*, respectively [60]. We identified both insertions in 1B-D1606 and 74-D694, but not in 6P-33G-D373, as the strain acquired additional wild-type copy of *LEU2* as a marker during integration of *PmSUP35* into *SUP35* locus [25] which probably renders both indels heterozygous.

LYS2

lys2-87 (*L28*) was obtained by UV irradiation in 2V-P3982 and was shown to be a TGA nonsense (see [17]). *LYS2* is a very long gene (over 4 kbp); that is probably why the PGC *LYS2* allele contains 17 substitutions, including three presumably cryptic missense mutations. All the 17 substitutions were detected in all the five strains analyzed. The only additional change was G3465A found in the 25-25-2V-P3982 *LYS2* allele, and, indeed, it leads to a TGA nonsense at codon 1155. We thus conclude that this substitution corresponds to *lys2-87*. It also allows us to conclude that 238 amino acid C-terminal truncation makes the protein non-functional.

LYS9

The *lys9-A21* mutation was obtained in the A21-15V-P4 auxotroph. It was mapped to chromosome XIV, shown to be allelic to *lys9* [17], and later was identified as an ochre nonsense mutation by Simarov *et al*. (see [58]). The allele from 1B-D1606 was sequenced [59]. In addition to the reported T605A substitution corresponding to the *lys9-A21*, we found three synonymous SNVs in all the strains except 74-D694, which, as in case of *HIS7*, probably bears the *LYS9* allele of a different origin.

PHA2

pheA10 mutation ascends to A10-15V-P4, an auxotrophic derivative of 15V-P4, which was shown to require phenylalanine for growth ([17] and L. Murashko, unpublished data). Earlier, phenylalanine auxotrophy mutation *pha2* was described by Mortimer and Hawthorne [66]. Even though mutants from R.K. Mortimer were used for allelism tests of other 15V-P4-derived auxotrophic mutants [17], allelism test for *pheA10* and *pha2* was not conducted. Interestingly, both works failed to link the respective mutations to known centromeric markers. *pheA10* was also reported to provide incomplete inhibition of growth in absence of phenylalanine ("leaky" auxotrophy) which additionally obstructed genetic analysis [17]. The marker was eventually inherited by the 33G-D373 strain that was extensively used to study nonsense suppression and the [*PSI*$^+$] factor [65, 146]. The nature of the corresponding mutation which is a TAA nonsense was then discovered, but it was not assigned to any particular gene ([61] and unpublished data). In this work we succeeded in localizing *pheA10* mutation to a *PHA2* locus. We looked for mutations in phenylalanine biosynthesis genes and found a variation in *PHA2* annotated as a one base pair long deletion (S6 Fig). However, manual inspection of short read alignment and subsequent Sanger resequencing of this region allowed us to find a substitution leading to appearance of a premature termination codon (PTC). What seemed to be an indel turned out to be two-nucleotide variation in a single codon. The substitution in the second position of codon 161 is a 15V-P4-derived Leu to Lys missense mutation, but combined with A to T substitution in the first position it leads to an in-frame TAA appearance. Since the Peterhof allele turned out to be differ greatly from the one in the reference strain, we designated Peterhof wild-type allele as *PHA2P* (P for the Peterhof allele) and recommend further usage of *pha2P-A10* instead of *pheA10* designation for the mutation.

THR4

An auxotrophic mutation *thr4-B15* was obtained in 4B-2B-(4-10). It was mapped to chromosome III (together with the mating type locus and *leu2*) [17] and later shown to be a TAA nonsense mutation [52]. The exact substitution leading to the mutation has never been identified. We found A1180T substitution unique for 25-25-2V-P3982 that resulted in a TAA appearance and thus corresponded to *thr4-B15.* The Peterhof alleles of *THR4* contain an additional missense mutation found in 15V-P4, 25-25-2V-P3982, and 74-D694.

TRP1

TAG nonsense mutation *trp1-289* [62] characterizes all the analyzed strains of hybrid origin as they inherited the allele from DBY747 (S1 Fig, [147]). Sequencing showed that the C403T (Gln135[TAG]) substitution was responsible for the trait [59]. We found the same substitution in all the *trp1-289* strains. Surprisingly, the wild type Peterhof allele of this gene (present in 15V-P4 and 25-25-2V-P3982) contains a C403A (Gln135Lys) substitution in exactly the same position, which is not present in any other SGD strain. No other PGC substitutions in this gene were detected.

URA3

25-25-2V-P3982 bears a complete deletion of the *URA3* gene (dU8), which was obtained in 132-L28-2V-P3982, and the resulting strain was designated dU8-132-L28-2V-P3982 (O. Tarunina, unpublished data). Almost no data remained about how this mutation had been obtained and which exact nucleotide positions had been affected. Copy number variation analysis confirmed the absence of *URA3* in the 25-25-2V-P3982 genome. Manual inspection of short read alignment allowed us to pinpoint the exact starting and ending positions of the deletion.

All the strains of the hybrid origin analyzed contain the *ura3-52* marker, which they inherited from DBY747 (S1 Fig). It was determined as a transposon insertion [63] and thus could not be revealed with SNV or CNV analyses. We queried the *de novo* assembled contigs with the sequences of 5' and 3' parts of the gene. In every case, we found these sequences in two different contigs, consistent with transposons being at breakpoints of the assembly. The sequences adjacent to the *URA3* fragments were analyzed with *S. cerevisiae* WU-BLAST2 Search [95] and found to be highly similar to Ty-like elements. We found that the 5-nucleotide motif, ranging 115-120, was duplicated, which is a common occurrence during transposon insertion and perfectly corroborates the known allele sequence [63].

Supplementary references

136. Breining P, Piepersberg W. Yeast omnipotent supressor *SUP1* (*SUP45*): nucleotide sequence of the wildtype and a mutant gene. Nucleic acids research. 1986;14(13): 5187-97.
137. Inge-Vechtomov S, Zhouravleva G, Philippe M. Eukaryotic release factors (eRFs) history. Biol Cell. 2003;95: 195–209.
138. Helsen CW, Glover JR. Insight into molecular basis of curing of [*PSI*$^+$] prion by overexpression of 104-kDa heat shock protein (Hsp104). Journal of Biological Chemistry. 2012;287(1): 542-56.
139. Kushnirov VV, Kochneva-Pervukhova NV, Chechenova MB, Frolova NS, Ter-Avanesyan MD. Prion properties of the Sup35 protein of yeast *Pichia methanolica*. The EMBO journal. 2000;19(3): 324-31.
140. Breining P, Surguchov AP, Piepersberg W. Cloning and identification of a DNA fragment coding for the *SUP1* gene of *Saccharomyces cerevisiae*. Current genetics. 1984;8(6): 467-70.
141. Simarov BV, Mironova LN, Inge-Vechtomov SG. Nonsense-missense suppression in yeast. Mol Gen Genet. 1971;113(4): 302-7.
142. Inge-Vechtomov SG, Simarov BV, Soidla TR, Kožin SA. Super-suppressor induced interallelic complementation. Z Vererbungsl. 1966;98(4): 375-84.
143. Kvasha VV, Gordenin DA, Inge-Vechtomov SG. [Distribution of noncomplementing and polarly complementing mutations within *ade2* locus in the yeast *Saccharomyces cerevisiae*.] Genetika. 1976: 126-138.
144. Leeds P, Wood JM, Lee BS, Culbertson MR. Gene products that promote mRNA turnover in *Saccharomyces cerevisiae*. Mol Cell Biol. 1992;12: 2165–77.
145. Hinnen A, Hicks JB, Fink GR. Transformation of yeast. PNAS USA. 1978;75(4): 1929-33.
146. Chernoff YO, Inge-Vechtomov SG, Derkach IL, Ptyushkina MV, Tarunina OV, Dagkesamanskaya AR, Ter-Avanesyan MD. Dosage-dependent translational suppression in yeast *Saccharomyces cerevisiae*. Yeast. 1992;8(7): 489-99.
147. Botstein D, Falco SC, Stewart SE, Brennan M, Scherer S, Stinchcomb DT, Struhl K, Davis RW. Sterile host yeasts (SHY): a eukaryotic system of biological containment for recombinant DNA experiments. Gene. 1979;8(1): 17-24.
